# Bayesian Multi-Group Gaussian Process Models for Heterogeneous Group-Structured Data

**Published:** 2025

**Authors:** Didong Li, Andrew Jones, Sudipto Banerjee, Barbara Engelhardt

**Affiliations:** Department of Biostatistics, University of North Carolina at Chapel Hill, Chapel Hill, NC 27599, USA; Department of Computer Science, Princeton University, Princeton, NJ 08540, USA; Department of Biostatistics, University of California, Los Angeles, Los Angeles, CA 90095, USA; Gladstone Institutes, San Francisco, CA 94158, USA; Department of Biomedical Data Science, Stanford University, Stanford, CA 94305, USA

**Keywords:** Mixed data, covariance functions, Gaussian processes, semiparametric regression

## Abstract

Gaussian processes are pervasive in functional data analysis, machine learning, and spatial statistics for modeling complex dependencies. Scientific data are often heterogeneous in their inputs and contain multiple known discrete groups of samples; thus, it is desirable to leverage the similarity among groups while accounting for heterogeneity across groups. We propose multi-group Gaussian processes (MGGPs) defined over $R^{p}\times 𝒞$, where 𝒞 is a finite set representing the group label, by developing general classes of valid (positive definite) covariance functions on such domains. MGGPs are able to accurately recover relationships between the groups and efficiently share strength across samples from all groups during inference, while capturing distinct group-specific behaviors in the conditional posterior distributions. We demonstrate inference in MGGPs through simulation experiments, and we apply our proposed MGGP regression framework to gene expression data to illustrate the behavior and enhanced inferential capabilities of multi-group Gaussian processes by jointly modeling continuous and categorical variables.

## 1. Introduction

Gaussian processes (GPs, Rasmussen and Williams (2005)) are widely used in modeling complex dependent data in diverse inferential settings including nonlinear regression (Ghosal and Van der Vaart, 2017), spatial statistics (Stein, 1999), classification problems (Bernardo et al., 1998) and, increasingly, in deep learning and reinforcement learning applications (Damianou and Lawrence, 2013; Deisenroth et al., 2013). A GP endows an uncountable collection of random variables with a probability law so that any finite subset is multivariate Gaussian. This is achieved through a real-valued positive definite function K:𝒳×𝒳→R, which acts as a covariance function or kernel.

We develop GPs for analyzing “multi-group” data, where measurements belong to one of k groups. Examples include biological measurements from distinct tissues or cell types (Consortium et al., 2020; Regev et al., 2017); geospatial data from multiple locations defined by discrete demarcations, such as state or country borders (Pan et al., 2020); and census data from people of different races, ethnicities, and genders (Bureau, 2020). Models built on Euclidean domains do not account for the discrete set of groups. While GPs on non-Euclidean manifolds and graphs have attracted recent attention (Niu et al., 2019; Dunson et al., 2020; Li et al., 2023) and machine learning (Borovitskiy et al., 2020, 2021), the multi-group setting remains largely unaddressed.

For flexibly modeling k-group data, we seek a stochastic process over $𝒳:R^{p}\times 𝒞$ to drive the inference, where $𝒞=\left\{c_{1},\cdots ,c_{k}\right\}$ is a finite set representing group labels. We specifically extend three existing approaches over Euclidean domains (Park and Choi, 2010): Separate Gaussian processes (SGPs), Union Gaussian processes (UGPs), and Hierarchical Gaussian processes (HGPs). The SGP assumes independence across groups. Therefore, the across-group correlation is set to zero: $K\left(\left(x,c_{i}\right),\left(x^{\prime },c_{j}\right)\right)=0$ if i≠j. The SGP is equivalent to modeling each group with a separate, independent GP. The UGP assumes the same dependencies within and across groups, so the covariance function does not depend on the members of 𝒞, i.e., $K\left(\left(x,c_{i}\right),\left(x^{\prime },c_{j}\right)\right)=K_{0}\left(x,x^{\prime }\right)$. It is equivalent to modeling all groups jointly with a single GP. The HGP accommodates both across- and within-group dependencies, where all within-group dependencies are assumed to be identical, and all across-group dependencies are assumed to be identical as well. Here, $K\left(\left(x,c_{i}\right),\left(x^{\prime },c_{j}\right)\right)=K_{0}\left(x,x^{\prime }\right)+1_{\left\{c_{i}=c_{j}\right\}}K_{1}\left(x,x^{\prime }\right)$, where $K_{0}$ and $K_{1}$ are real-valued positive definite functions (Park and Choi, 2010; Hensman et al., 2013). Each of the above models build covariance functions based upon a standard GP over a Euclidean domain. These models are often used in practice for their simplicity and extend beyond the context of GPs (Tsherniak et al., 2017). However, each of these three GPs types impose restrictive conditions and fail to model heterogeneous between-group dependencies. If the model is misspecified, the inferential performance of these processes will be unsatisfactory, especially when the overall sample size is small or the groups are imbalanced in terms of sample size.

We introduce multi-group Gaussian process (MGGP) models (Section 2) through the construction of positive definite covariance functions over $𝒳≔R^{p}\times 𝒞$ (Section 3). The multi-group process flexibly models heterogeneity across and within groups, leveraging varying levels of similarity between groups and allows us to exploit prior or expert knowledge. Since the multi-group structure is encoded in the covariance function, we can use existing methods and computational algorithms for fitting standard GP models.

The MGGP contributes to the literature on joint modeling of continuous and categorical variables (Dunson et al., 2003; Dunson, 2000; Teimourian et al., 2015; Ru et al., 2020; Schulam et al., 2015; Murray and Reiter, 2016; Leroy et al., 2022, 2023) by avoiding additive, hierarchical, and mixture models entirely. Instead, the MGGP explicitly modeling dependencies within and among the continuous and categorical variables. This flexibility allows straightforward conditioning on multiple categorical partitions, offering a tractable conditional posterior, and enables us to exploit dependencies between groups when some groups have small sample sizes. The inferential benefits of our approach are illustrated using maximum likelihood and full Bayesian inference through simulation experiments and an analysis of gene expression data (Section 4). We conclude with some remarks in Section 6. The Appendix includes proofs of theoretical results, code, data and additional analysis.

## 2. Multi-Group Gaussian Process Regression Models

We consider a dependent variable $y\left(x;c_{j}\right)$ generated from a latent stochastic process over $R^{p}\times 𝒞$ for inputs $x\in R^{p}$ and group j through the model

(1) $$y\left(x;c_{j}\right)=\mu \left(x;c_{j}\right)+Z\left(x;c_{j}\right)+\epsilon \left(x;c_{j}\right),\;\epsilon \left(x;c_{j}\right)\overset{ind}{\sim }N\left(0,\tau_{j}^{2}\right),$$

where $\mu \left(x;c_{j}\right)$ is a mean function, $Z\left(x;c_{j}\right)$ is a zero-centered latent process, and $\epsilon \left(x;c_{j}\right)$ is a zero-centered white-noise process capturing measurement error or fine-scale variation with group-specific variances. The mean function can be further modeled, if appropriate, as $\mu \left(x;c_{j}\right)=f_{j}(x{)}^{\text{T}}\beta_{j}$, where $f_{j}(x)$ is a $q_{j}\times 1$ vector of design variables possibly, but not necessarily, depending on x, and each $\beta_{j}$ is a $q_{j}\times 1$ vector of group-specific regression coefficients. This specification accommodates predictors or other explanatory variables that need neither be continuous nor reside within $R^{p}$.

Equation (1) includes a parametric specification through the mean function and a nonparametric specification through the latent process. Our focus in this paper is not so much on modeling $\mu \left(x;c_{j}\right)$, which can be built from standard linear model specifications, as it is on $Z\left(x;c_{j}\right):𝒳⟶R$, where $𝒳=R^{p}\times 𝒞$. We will specify Z(⋅;⋅) to be a GP with zero mean and covariance function $K\left(\left(x;c_{j}\right),\left(x^{\prime };c_{j^{\prime }}\right)\right):𝒳\times 𝒳⟶R$ so that $K\left(\left(x;c_{j}\right),\left(x^{\prime };c_{j^{\prime }}\right)\right)=\text{cov}\left(Z\left(x;c_{j}\right),Z\left(x^{\prime };c_{j^{\prime }}\right)\right)$ is a positive-definite covariance function. We consider settings where data arise over a finite, possibly imbalanced, set of points $\left\{\left(x_{i};c_{j}\right)\right\}$ for $i=1,2,\ldots ,n_{j}$ and j=1,2,…,k. Each group can have a different number, $n_{j}$, of inputs. Given the covariance function, the realizations of the process over the finite set of points is the n×1 vector $Z={\left(Z_{1}^{\text{T}},\ldots ,Z_{k}^{\text{T}}\right)}^{\text{T}}$, where $n=\sum_{j=1}^{k}n_{j}$ and $Z_{j}={\left(Z\left(x_{1};c_{j}\right),\ldots ,Z\left(x_{n_{j}};c_{j}\right)\right)}^{\text{T}}$ follows a multivariate Gaussian distribution with an n×1 zero vector as the mean and an n×n covariance matrix K, whose $\left(j,j^{\prime }\right)$ th block is given by the $n_{j}\times n_{j^{\prime }}$ matrix $K_{jj^{\prime }}$ with $\left(i,i^{\prime }\right)$ element $K\left(\left(x_{i};c_{j}\right),\left(x_{i^{\prime }};c_{j^{\prime }}\right)\right)$ for $i=1,2,\ldots ,n_{j}$ and $i^{\prime }=1,2,\ldots ,n_{j^{\prime }}$.

Equation (1) enables likelihood-based inference and can be extended to a Bayesian hierarchical framework (Cressie and Wikle, 2011; Banerjee et al., 2014). Assuming, for elucidation purposes only, that $\mu \left(x;c_{j}\right)=f_{j}(x{)}^{\text{T}}\beta_{j}$, a Bayesian model specifies the joint distribution

(2) $$p\left(\left\{\tau_{j}^{2}\right\},\theta ,\left\{\beta_{j}\right\}\right)\times N\left(Z|0,K_{\theta }\right)\times \overset{k}{\underset{j=1}{\prod }}\overset{n_{j}}{\underset{i=1}{\prod }}N\left(y\left(x_{i};c_{j}\right)|f_{j}{\left(x_{i}\right)}^{\text{T}}\beta_{j}+Z\left(x_{i};c_{j}\right),\tau_{j}^{2}\right),$$

where θ denotes parameters in the covariance function, and $p\left(\left\{\tau_{j}^{2}\right\},\theta ,\left\{\beta_{j}\right\}\right)$ is the prior on the model parameters. Inference on these parameters and the latent process Z(⋅) proceeds by drawing samples from the posterior distribution $p\left(\left\{\tau_{j}^{2}\right\},\theta ,\left\{\beta_{j}\right\},Z|\left\{y\left(x_{i};c_{j}\right)\right\},\left\{f_{j}\left(x_{i}\right)\right\}\right)$, which is proportional to Equation (2).

Sampling from the joint posterior distribution including the process realizations Z will be challenging due to the dimension of Z. Exploiting the Gaussian likelihood, we work with the collapsed likelihood after integrating out Z from Equation (2), which yields

(3) $$p(\tau ,\theta ,\beta |y,F)\propto p(\tau ,\theta ,\beta )\times N\left(y|F\beta ,K_{\theta }+D_{\tau }\right),$$

where y is the n×1 vector of observations, $y\left(x_{i};c_{j}\right)$, constructed analogous to Z,F is an n×q block-diagonal matrix, $q=\sum_{j=1}^{k}q_{j}$, with $n_{j}\times q_{j}$ blocks $F_{j}={\left(f_{j}\left(x_{1}\right),\ldots ,f_{j}\left(x_{n_{j}}\right)\right)}^{\text{T}},\;\beta ={\left(\beta_{1}^{\text{T}},\ldots ,\beta_{k}^{\text{T}}\right)}^{\text{T}}$ is the q×1 vector of stacked regression coefficients, $\tau =\left\{\tau_{j}^{2}\right\}$ is the collection of error variances, and $D_{\tau }$ is the n×n diagonal matrix with $\tau_{j}^{2}I_{n_{j}}$ as $n_{j}\times n_{j}$ diagonal blocks. Markov chain Monte Carlo (MCMC) algorithms sample more efficiently from Equation (3) because of the reduced parameter space relative to Equation (2).

We sample from p(Z∣y,F)=E[p(Z∣{τ,θ,β},y,F)] to carry out inference on the latent process, where the expectation E[⋅] is taken with respect to the posterior distribution in Equation (3); we draw one Z~p(Z∣{τ,θ,β},y,F) for each posterior drawn value of {τ,θ,β}. This is straightforward because p(Z∣{τ,θ,β},y,F) is of the form N(Mm,M), where $M^{-1}=K_{\theta }^{-1}+D_{\tau }^{-1}$ and m=y-Fβ, and the draws need to be made using only the post-convergence samples of {τ,θ,β}.

To estimate the latent process at an unobserved input $x_{0}\in R^{p}$ for a given group $c_{j}\in 𝒞$, we evaluate the Bayesian posterior predictive distribution

(4) $$p\left(Z\left(x_{0};c_{j}\right)|\left\{y\left(x_{i};c_{j}\right)\right\},\left\{f_{j}\left(x_{i}\right)\right\}\right)\propto \int p\left(Z\left(x_{0};c_{j}\right)|Z,\theta \right)\times p(Z,\{\tau ,\theta ,\beta \}|y,F)dZd\{\tau ,\theta ,\beta \},$$

where we use the conditional independence $p\left(Z\left(x_{0};c_{j}\right)|Z,\{\tau ,\theta ,\beta \},y,F\right)=p\left(Z\left(x_{0};c_{j}\right)|Z,\theta \right)$ derived from the hierarchical model in Equation (2). We sample from Equation (4) by drawing one $Z\left(x_{0};c_{j}\right)\sim p\left(Z\left(x_{0};c_{j}\right)|Z,\theta \right)$ for each drawn posterior sample of Z and θ, where $p\left(Z\left(x_{0};c_{j}\right)|Z,\theta \right)$ is Gaussian with mean $K_{\theta }{\left(\left(x_{0};c_{j}\right);\cdot \right)}^{\text{T}}K_{\theta }^{-1}Z,K_{\theta }\left(\left(x_{0};c_{j}\right);\cdot \right)$ is the n×1 vector with elements $K_{\theta }\left(\left(x_{0};c_{j}\right),\left(x_{i},c_{j^{\prime }}\right)\right)$ for $j^{\prime }=1,\ldots ,k$ and $i=1,2,\ldots ,n_{j^{\prime }}$, and variance $K_{\theta }\left(\left(x_{0};c_{j}\right),\left(x_{0};c_{j}\right)\right)-K_{\theta }{\left(\left(x_{0};c_{j}\right);\cdot \right)}^{\text{T}}K_{\theta }^{-1}K_{\theta }\left(\left(x_{0};c_{j}\right);\cdot \right)$. To predict $Y\left(x_{0};c_{j}\right)$, we sample from the predictive distribution $p\left(Y\left(x_{0};c_{j}\right)|y,F\right)$ by drawing one $Y\left(x_{0};c_{j}\right)\sim N\left(f_{j}{\left(x_{0}\right)}^{\text{T}}\beta_{j}+Z\left(x_{0};c_{j}\right),\tau_{j}^{2}\right)$ for each posterior sample of $\left\{\beta_{j},\tau_{j}^{2}\right\}$ and $Z\left(x_{0};c_{j}\right)$.

We need valid positive-definite functions to serve as $K_{\theta }\left(\left(x;c_{j}\right),\left(x^{\prime };c_{j^{\prime }}\right)\right)$. This is crucial for the above inferential framework as it ensures that the matrix $K_{\theta }$ in Equation (2) will be positive definite for any finite set of distinct elements, observed or unobserved, in R×𝒞. An advantage of driving the inference through a latent process is the convenience of predictive inference for the underlying process and the response at new inputs. Therefore, we focus upon the construction of valid covariance functions to specify MGGPs.

The computational bottleneck arises from the dimension of $K_{\theta }$ in GP models for large data sets. There is a substantial literature on various approaches to build models that scale up to massive data sets by building low-rank or sparsity-inducing processes (see, e.g. Wikle, 2010; Banerjee, 2017; Heaton et al., 2019, for expository treatments) from any valid covariance function. While our current focus is not specifically on processes that scale inference to massive data sets, we note that constructing MGGPs using valid covariance functions renders the resulting processes as “scalable-ready” since low-rank or sparsity-inducing variants may be derived using existing approaches.

## 3. Multi-Group Gaussian Processes

Proofs of all theoretical results presented below are in the Appendix.

### 3.1 Separable multi-group GPs

We start with a simple case where the covariance function over $R^{p}\times 𝒞$ is separable.

**Definition 1 K** *is said to be separable if* $K\left(\left(x,c_{i}\right),\left(x^{\prime },c_{j}\right)\right)=K_{R^{p}}\left(x,x^{\prime }\right)K_{𝒞}\left(c_{i},c_{j}\right)$, *where* $K_{R^{p}}$ *and* $K_{𝒞}$ *are over* $R^{p}$ *and* 𝒞, *respectively*.

Note that K is positive definite if and only if both $K_{R^{p}}$ and $K_{𝒞}$ are positive definite. Constructing valid GPs over $R^{p}$ is well known, so we focus on covariance functions $K_{𝒞}$, i.e., GPs over a categorical set. Also, 𝒞 being finite, any function on 𝒞×𝒞 is completely determined by the k×k positive definite matrix C with elements $C_{ij}=K_{𝒞}\left(c_{i},c_{j}\right)$.

**Proposition 2** $K_{𝒞}$ *is positive definite if and only if* C *is a positive definite matrix*.

Thus, a positive definite function on $R^{p}$ and a positive definite matrix $C\in R^{k\times k}$ ensures a separable positive definite function on 𝒳. Homogeneous kernels arise as a special case.

**Definition 3** *A function* $K_{𝒞}:𝒞\times 𝒞\to R$ *is said to be homogeneous if* $K_{𝒞}\left(c_{i},c_{j}\right)=K_{0}\left(1_{\left\{c_{i}\neq c_{j}\right\}}\right)$ *for some function* $K_{0}$ *on* {0, 1}.

A homogeneous process is completely determined by two scalars $a≔K_{𝒞}\left(c_{i},c_{i}\right),b≔K_{𝒞}\left(c_{i},c_{j}\right)$ with $c_{i}\neq c_{j}$, which represent the within-group and across-group associations, respectively. Without loss of generality, we assume a=1; otherwise, we can rescale $K_{𝒞}$. In this case, both within-group and between-group correlations are constants. A homogeneous process is appropriate if we only want to distinguish pairs of observations in the same group from those in different groups, while the specific group identities are irrelevant. Hence, K is homogeneous if it is isotropic with respect to the discrete metric $d\left(c_{i},c_{j}\right)=1_{\left\{c_{i}\neq c_{j}\right\}}$.

**Corollary 4** *Let* $K_{𝒞}$ *be homogeneous, then* $K_{𝒞}$ *is positive definite if and only if* $-\frac{1}{k-1}\leq b\leq 1$, *where* $b=K_{𝒞}\left(c_{i},c_{j}\right)$ *with* i≠j.

The inequality $-\frac{1}{k-1}\leq b\leq 1$ implies that across-group correlations should not dominate the within-group correlations, which is intuitively reasonable. Separable models provide computational benefits because the resulting covariance matrix for Z can be expressed as a Kronecker product $K_{R}⊗K_{𝒞}$. However, such covariance functions tend to have “ridges” or discontinuities (Stein, 2005) that lead to worse inference. They also assume that the same covariance structure $\left(K_{R^{p}}\right)$ is retained for all groups, which is restrictive in terms of accommodating associations for different pairs of inputs in $R^{p}\times 𝒞$.

### 3.2 Isotropic multi-group GPs

In order to discuss “isotropic” covariance functions on $R^{p}\times 𝒞$, we endow 𝒞 with additional structure. To facilitate our development, we introduce a metric d on 𝒞 so that (𝒞,d) is a metric space.

**Definition 5** *Given two metric spaces* (𝒴,d) *and* $\left({𝒴}^{\prime },d^{\prime }\right)$, *a* GP *on* $𝒴\times {𝒴}^{\prime }$ *is said to be semi-isotropic if* $K\left(\left(x_{1},x_{1}^{\prime }\right),\left(x_{2},x_{2}^{\prime }\right)\right)=K_{0}\left(d\left(x_{1},x_{2}\right),d^{\prime }\left(x_{1}^{\prime },x_{2}^{\prime }\right)\right)$.

Intuitively, a semi-isotropic process is isotropic in $R^{d}$ and 𝒞 separately. Isotropy implies semi-isotropy, but the other direction does not hold in general. In practice, d is usually obtained from domain knowledge including prior, exterior, or expert knowledge. Thus, if 𝒞={NorthCarolina,NewJersey,California}, then the distance can be the geographical distance between the centroids of these states. As another example, if 𝒞 represents human tissue types, then d can be constructed using prior biomedical knowledge; two tissue types from the same organ (e.g., brain) might tend to be more similar to each other than from different organs (e.g., brain and liver). If 𝒞 is a weighted graph, then the graph distance serves as a valid metric (Bouttier et al., 2003). If domain knowledge is unavailable, a default noninformative distance, $d_{ij}=1-\delta_{ij}$, implying that all groups are equidistant, can be adopted. Extensions to unknown $d_{ij}$, which are instead treated as parameters, are discussed in Appendix I. Our next result creates a large family of semi-isotropic covariance functions on $𝒳=R^{p}\times 𝒞$.

**Theorem 6** *Assume the Gram matrix defined as $G_{ij}≔\frac{1}{2}\left(d\left(c_{1},c_{i}\right)+d\left(c_{1},c_{j}\right)-d\left(c_{i},c_{j}\right)\right)$ is positive semi-definite, then if* $\varphi :R_{+}\to R$ *is a completely monotone function and* $\psi \;:\;R_{+}\to R_{+}$ *is a positive function with a completely monotone derivative, then:*

(5) $$K\left(\left(x,c_{i}\right),\left(x^{\prime },c_{j}\right)\right)=\frac{\sigma^{2}}{{\left(\psi \left(d_{ij}^{2}\right)\right)}^{p/2}}\varphi \left(\frac{{\left\|x-x^{\prime }\right\|}^{2}}{\psi \left(d_{ij}^{2}\right)}\right).$$

*is a valid covariance function, where* $\sigma^{2}>0$ *is the spatial variance. In particular, if* $d\left(c_{i},c_{j}\right)=1-\delta_{ij}$ *is the discrete metric, then*

(6) $$K\left(\left(x,c_{i}\right),\left(x^{\prime },c_{j}\right)\right)=\frac{\sigma^{2}}{\alpha^{\frac{p}{2}\left(1-\delta_{ij}\right)}}\varphi \left(\frac{{\left\|x-x^{\prime }\right\|}^{2}}{\alpha^{1-\delta_{ij}}}\right)$$

*is a valid covariance function, where* $\sigma^{2}\in (0,\;1]$ *is the spatial variance and* α>0 *controls the interaction between* $R^{p}$ *and* 𝒞.

A simple form for G emerges when $d\left(c_{i},c_{j}\right)=1-\delta_{ij}$. The resulting Gram matrix is $G=\left(\begin{array}{cc} 0 & 0_{1\times (k-1)}\\ 0_{(k-1)\times 1} & \tilde{G} \end{array}\right)$, where $\tilde{G}=\frac{1}{2}{\text{Id}}_{k-1}+\frac{1}{2}1_{\left(k-1)\times (k-1\right)}$ and $1_{m\times n}$ denotes the m×n matrix of ones. Some candidates for completely monotone functions ϕ and positive functions with completely monotone derivatives ψ are in Table 1 (Gneiting, 2002). This class of covariance functions is also known as the “Gneiting class”. Selecting ϕ and ψ from Table 1, we obtain the following semi-isotropic covariance functions on 𝒳 (for more covariance functions, see Section H):

(7) $$K\left(\left(x,c_{i}\right),\left(x^{\prime },c_{j}\right)\right)=\frac{\sigma^{2}}{{\left(a^{2}d_{ij}^{2}+1\right)}^{p/2}}\text{exp}\left\{-\frac{b^{2}{\left\|x-x^{\prime }\right\|}^{2}}{a^{2}d_{ij}^{2}+1}\right\},$$

(8) $$K\left(\left(x,c_{i}\right),\left(x^{\prime },c_{j}\right)\right)=\left\{\begin{array}{ll} \frac{\sigma^{2}2c^{p/2}}{{\left(a^{2}d_{ij}^{2}+1\right)}^{\nu }{\left(a^{2}d_{ij}^{2}+c\right)}^{p/2}\Gamma \left(\nu \right)}{\left\{\frac{b}{2}{\left(\frac{a^{2}d_{ij}^{2}+1}{a^{2}d_{ij}^{2}+c}\right)}^{1/2}\left\|x-x^{\prime }\right\|\right\}}^{\nu } & \\ \times K_{\nu }\left(b{\left(\frac{a^{2}d_{ij}^{2}+1}{a^{2}d_{ij}^{2}+c}\right)}^{1/2}\left\|x-x^{\prime }\right\|\right) & x\neq x^{\prime }\\ \frac{\sigma^{2}c^{p/2}}{{\left(a^{2}d_{ij}^{2}+1\right)}^{\nu }{\left(a^{2}d_{ij}^{2}+c\right)}^{p/2}} & x=x^{\prime } \end{array}\right.,$$

(9) $$K\left(\left(x,c_{i}\right),\left(x^{\prime },c_{j}\right)\right)=\frac{\sigma^{2}c^{p/2}}{{\left(a^{2}d_{ij}^{2}+1\right)}^{1/2}{\left(a^{2}d_{ij}^{2}+c\right)}^{p/2}}\text{exp}\left\{-b{\left(\frac{a^{2}d_{ij}^{2}+1}{a^{2}d_{ij}^{2}+c}\right)}^{1/2}\left\|x-x^{\prime }\right\|\right\}.$$

In the above functions, $\sigma^{2}>0$ is the spatial variance, a≥0 is the group similarity scale, b≥0 is the feature scale, c≥0 is the separability scale, and ν>0 is a smoothness parameter. The covariance function in Equation (7) is analogous to the squared exponential or radial basis functions (RBFs). The covariance function in Equation (8) is the analogue of the Matérn covariance function. In particular, the covariance function in Equation (9) is a special case of Equation (8) when ν=1/2, which is the exponential covariance function. The covariance function in Equation (8) becomes separable when c=1. We supply a table summarizing the kernel constructions in Appendix H.

It is important to clarify that ϕ and ψ are legitimate choices within the Gneiting class, and modelers can select one independent of the other from the provided list. The final decision on these parameters should be based on the specific problem at hand. We also remark that our proposed framework is broader than the Gneiting class, and we do not imply that the Gneiting class encompasses all possible choices. Appendix H provides more examples of kernels within and beyond the Gneiting class.

### 3.3 Stationary multi-group GPs

We now weaken isotropy. Since (𝒞,d) does not admit a natural algebraic structure, we start with k=2, which appears frequently in practice including in data sets where the two groups are male/female, adults/children, treatment/control, and so on. For the nonisotropic case, 𝒞 can be identified with $Z_{2}$ when k=2, an Abelian group. In this setting, K is said to be stationary if $K\left((x,d),\left(x^{\prime },l\right)\right)=K_{0}\left(x-x^{\prime },d-l\right)$. We use K instead of $K_{0}$ for simplicity, where K is characterized by $K_{w}=K(\cdot ,0)$, the within-group covariance function, and $K_{c}=K(\cdot ,1)$, the cross-group covariance function. Any covariance function on $R^{p}\times Z_{2}$ determines two covariance functions on $R^{p}$. On the other hand, not all pairs of covariance functions on $R^{p}$ define a valid covariance function on $R^{p}\times Z_{2}$. In order to construct a valid covariance function on $R^{p}\times Z_{2}$, we need a sufficient condition for K to be positive definite.

**Theorem 7** *Let* $K_{w}$ *and* $K_{c}$ *be two positive definite functions on* $R^{p}$ *with spectral densities* $\rho_{w}$ *and $\rho_{c}$ such that* $K(x,0)=K_{w}(x)=\int_{R^{p}}e^{-2\pi i\omega x}\rho_{w}(\omega )d\omega ,\;K(x,1)=K_{c}(x)=\int_{R^{p}}e^{-2\pi i\omega x}\rho_{c}(\omega )d\omega$. *Then*, $K(x,l)=\left\{\begin{array}{ll} K_{w}(x) & l=0\\ K_{c}(x) & l=1 \end{array}\right.$ *is positive definite on* $R^{p}\times Z_{2}$ *if and only if* $\rho_{w}\geq \rho_{c}$.

**Example 1** *Recall the multi-group RBF in* Equation (7). *The two spectral densities are $\rho_{w}(\omega )=\sigma^{2}{\left(\frac{\pi }{b^{2}}\right)}^{\frac{p}{2}}\text{exp}\;\left\{-\frac{\pi^{2}\|\omega {\|}^{2}}{b^{2}}\right\}$ and* $\rho_{c}(\omega )=\sigma^{2}{\left(\frac{\pi }{b^{2}}\right)}^{\frac{p}{2}}\text{exp}\;\left\{-\frac{\pi^{2}\left(a^{2}+1\right)\|\omega {\|}^{2}}{b^{2}}\right\}$, *where* $\rho_{w}\geq \rho_{c}$.

The stationary MGGP assumes homogeneity in the within-group correlation. To account for heterogeneity, we introduce a weaker semi-stationary MGGP. This semi-stationary process is stationary in $R^{p}$ but not in 𝒞.

**Definition 8** K *is said to be semi-stationary if* $K\left(\left(x,c_{i}\right),\left(x^{\prime },c_{j}\right)\right)=K_{0}\left(x-x^{\prime },c_{i},c_{j}\right)$ *where* $K_{0}$ *is defined on* $R^{p}\times 𝒞\times 𝒞$.

A semi-stationary process is appropriate for applications where groups are expected to have different within-group correlations, but the process is stationary once the group is fixed. For semi-stationary MGGPs, K is determined by $K_{0}(x)=K(x,0,0),K_{c}(x)=K(x,0,1)=K(x,1,0)$ and $K_{1}=K(x,1,1)$, where $K_{0}\neq K_{1}$ in general; otherwise K becomes stationary.

**Theorem 9** *Let* $K_{0},\;K_{c}$ *and $K_{1}$ be positive definite functions on* $R^{p}$ *with spectral densities $\rho_{0},\;\rho_{c}$*, *and* $\rho_{1}$. Then $K\left(x,l,l^{\prime }\right)=\left\{\begin{array}{ll} K_{0}(x) & l=l^{\prime }=0\\ K_{c}(x) & l+l^{\prime }=1\\ K_{1}(x) & l=l^{\prime }=1 \end{array}\right.$ *is positive definite on* $R^{p}\times Z_{2}$ *if and only if* $\rho_{0}\rho_{1}\geq \rho_{c}^{2}$.

Data sets with more than two groups are ubiquitous in scientific applications. Hence, we generalize the above theory to k>2 groups. The difficulty here is that 𝒞 does not admit a natural group structure for k>2. A straightforward solution would be to identify 𝒞 with $Z_{k}$, but the modular structure of $Z_{k}$, i.e., 1-0=2-1=⋯k-1-(k-1-1)≠k-(k-1), is not satisfied in practice. Hence, Bochner’s Theorem (Rudin, 2017), which characterizes positive definite functions on locally compact Abelian groups, is not applicable. Theorem 9 draws an equivalence between Bochner’s Theorem on $R^{p}\times Z_{2}$ and Cramér’s Theorem on $R^{p}$ with k=2. Hence, we can use bivariate GPs to construct two-group GPs. Furthermore, given that Cramér’s Theorem (Cramér, 1940) holds for a general k-variate GP, we can develop a general theory for an arbitrary number of groups with k>2, which we do below. We draw similarities between the MGGP with k groups and k-variate GPs, also known as multi-task GPs. Recall that a k-variate random field $\tilde{Z}$ on 𝒴 is characterized by its cross-covariance function $\tilde{K}:𝒴\times 𝒴\to R^{k\times k}:\text{cov}\left(\tilde{Z}(x),\tilde{Z}\left(x^{\prime }\right)\right)=\tilde{K}\left(x,x^{\prime }\right)$.

**Theorem 10** *Let* 𝒢 *be the space of all Gaussian random fields on* 𝒴×𝒞, *where* $𝒞=\left\{c_{1},\cdots ,c_{k}\right\}$ *and* 𝒱 *is the space of all Gaussian* k-*variate random fields on* 𝒴. *Then* $\Phi :𝒢\to 𝒱,(\Phi (Z){)}_{i}(x)≔Z\left(x,c_{i}\right),\forall Z\in 𝒢$ *is a bijection, and its inverse* $\Phi^{-1}$ *is given by* $\Phi^{-1}:𝒱\to 𝒢,\left(\Phi^{-1}(\tilde{Z})\right)\left(x,c_{i}\right)={\tilde{Z}}_{i}(x),\forall \tilde{Z}\in 𝒱$. *The correspondence between the covariance function of* Z *and the cross-covariance function of $\tilde{Z}$ is given by* $K\left(\left(x,c_{i}\right),\left(x^{\prime },c_{j}\right)\right)=\tilde{K}{\left(x,x^{\prime }\right)}_{ij}$.

Therefore, constructing a k-variate GP will produce a k-group GP, and vice versa. Existing constructions of multivariate GPs can be applied (see, e.g., Cressie and Huang, 1999; Gneiting et al., 2010; Apanasovich and Genton, 2010; Gelfand and Banerjee, 2010; Genton and Kleiber, 2015). While MGGPs and multi-task GPs are mathematically equivalent, they focus on different aspects of statistical learning. The multi-task GP focuses on predicting multiple tasks simultaneously by borrowing information across groups. On the other hand, the MGGP models multiple groups that may or may not have shared underlying structure by learning the kernel parameters explicitly. We prove the following related result.

**Theorem 11** *Let* $K:R^{p}\times 𝒞\times 𝒞\to R$ *be a function with* $K_{ij}=K\left(\cdot ,c_{i},c_{j}\right)$ *being stationary on $R^{p}$ and spectral densities* $\rho_{ij}$. *Then,* K *is positive definite, hence defines a semi-stationary GP on* $R^{p}\times 𝒞$, *if and only if* $\rho (\omega )=\{\rho (\omega ){\}}_{i,j=1}^{k}$ *is positive semi-definite for any* $\omega \in R^{p}$.

Theorem 9 is a special case of Theorem 11 when k=2, but can be proved differently. As a result, the connection between Bochner’s Theorem on $R^{p}\times 𝒞$ and Cramér’s Theorem on $R^{p}$ is analogous to the relationship between multi-group and multivariate GPs. The MGGP is a non-trivial generalization of existing processes that allows substantial group heterogeneity by accommodating a variety of flexible covariance functions.

### 3.4 Multivariate multi-group Gaussian processes

The construction of the MGGP can be extended to multivariate, or multi-output GPs. Let Z be a $k^{\prime }$-variate GP on $R^{p}\times 𝒞$ with cross-covariance function $K:R^{p}\times 𝒞\times R^{p}\times 𝒞\to R^{k^{\prime }\times k^{\prime }}$, that is, $\text{cov}\left(Z\left(x,c_{i}\right),Z\left(x^{\prime },c_{j}\right)\right)=K\left(\left(x,c_{i}\right),\left(x,c_{j}\right)\right)$. Similar to the construction of the MGGP, first we assume that there exists a metric $d^{\prime }$ between output variables with a positive semi-definite Gram matrix.

**Theorem 12** *If* $\varphi :R_{+}\to R$ *is a completely monotone function and* $\psi_{1},\psi_{2}:R_{+}\to R_{+}$ *are positive functions with completely monotone derivatives, then*

$$K{\left(\left(x,c_{i}\right),\left(x^{\prime },c_{j}\right)\right)}_{kl}=\frac{\sigma^{2}}{{\left(\psi_{1}\left(\frac{d_{ij}^{2}}{\psi_{2}\left(d_{kl}^{\prime 2}\right)}\right)\right)}^{p/2}{\left(\psi_{2}\left(d_{kl}^{\prime 2}\right)\right)}^{1/2}}\varphi \left(\frac{{\left\|x-x^{\prime }\right\|}^{2}}{\psi_{1}\left(\frac{d_{ij}^{2}}{\psi_{2}\left(d_{jl}^{\prime 2}\right)}\right)}\right)$$

*is a valid cross-covariance function, where* $\sigma^{2}>0$ *is the spatial variance*.

## 4. Simulations

### 4.1 Comparing the MGGP with related models on simulations

We conducted an experiment to assess the MGGP’s ability to recover the Separate, Union, and Hierarchical Gaussian processes as special cases. We generated data from each of these models using Equation (1) with k=2 groups. We specified a zero mean, i.e., $\mu \left(x;c_{j}\right)=0$ for both groups, and specified the latent process using covariance functions for the three models. We set $b=\sigma^{2}=a=1$ in Equation (7). (Note that a is only used in the generation of data from the MGGP.) We also assumed $\tau_{1}^{2}=\tau_{2}^{2}=\tau^{2}$ in Equation (1) and used $\tau^{2}=0.1$ to generate our data. Using these settings, we generated $n_{1}=n_{2}=100$ measurements for each group.

We computed the log marginal likelihood of the data, i.e., $N\left(y|0,K_{\theta }+D_{\tau }\right)$, under each model for each data set. For the SGP, UGP and HGP, we used the RBF, $K\left(x,x^{\prime }\right)=\sigma^{2}\;\text{exp}\left\{-b^{2}{\left\|x-x^{\prime }\right\|}^{2}\right\}$. For the Multi-Group model we used the “multi-group” RBF in Equation (7). When computing the likelihood under each model, we fix $b,\;\sigma^{2}$, and $\tau^{2}$ to their true values; for a we use a grid of values, $a=10^{-5},\;10^{-4},\ldots ,10^{2}$, and we specify $D_{\tau }=\tau^{2}I_{n}$.

**Figure 1:**
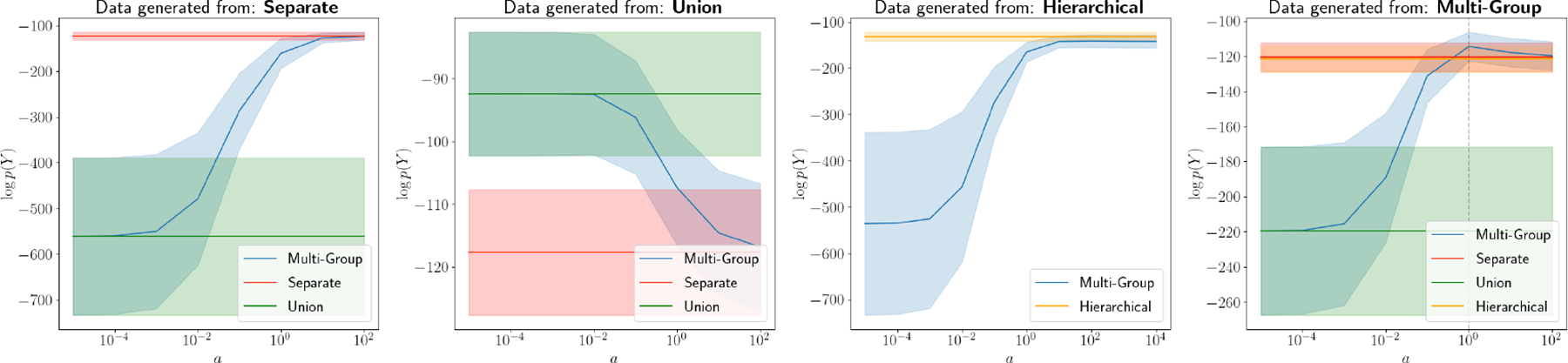
Comparison between the *Multi-Group, Separate, Union*, and *Hierarchical* process models. Using two-group data generated from each of the four models, we computed the log marginal likelihood of the data under each model. For the Multi-Group model, we used the covariance function in Equation (7) and used a range of different values the parameter a. In the rightmost plot, the dashed vertical line indicates the true value of a used for data generation. We used an RBF kernel, which does not have an a parameter, for the *Separate* and *Union* models. We repeated this experiment 20 times, and the bands in each plot represent 95% confidence intervals.

Our MGGP performs on par with the SGP, UGP and HGP in the expected regimes (Figure 1). The MGGP matches the performance (as measured by the log marginal likelihood) of the SGP when a is large, and the MGGP matches the performance of the UGP as a→0. For data generated from the MGGP, we find that the likelihood peaks at the true value of a and is higher than all other models at this value. These results i) serve as a demonstration of the role of a; ii) confirm numerically that the MGGP recovers these alternative models in certain regimes; and iii) suggest that the MGGP is a viable generalization of the other three models.

### 4.2 Estimation and inference for the MGGP

In our previous experiment, we used the multi-group covariance function in Equation (7) with a fixed value of a. In practice, we will need to estimate a and all other covariance parameters from the data. Next, we assess the parameter estimates of the MGGP using both maximum likelihood and fully Bayesian posterior inference.

**Figure 2:**
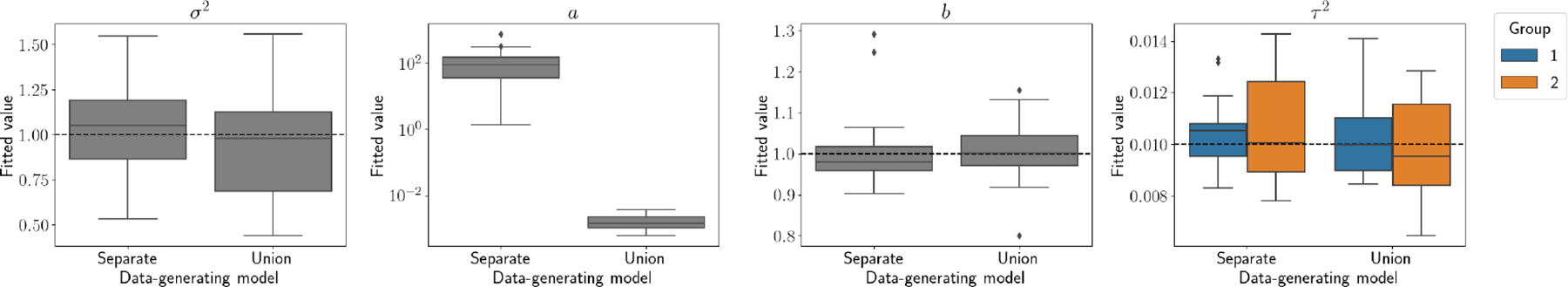
Covariance function parameter estimation. Using data generated from the Separate and Union processes, we fit the Multi-Group process by finding the MLEs for the true parameters of the kernel function in Equation (7). The boxes cover the interquartile range; the lower border of the box is the 25th percentile, the upper border of the box is the 75th percentile, and the middle line is the median. The whiskers extend 1.5 times the interquartile range in each direction.

We first conducted an experiment where we generated data from the SGP and UGP as in the previous section. We maximize the collapsed or marginalized likelihood corresponding to Equation (1), i.e., $𝒩\left(y|0,K_{\theta }+\tau^{2}I_{n}\right)$, with respect to $\theta =\left\{a,b,\sigma^{2}\right\}$, and a common measurement error variance $\tau^{2}$, where θ corresponds to the three parameters in the multi-group covariance function in Equation (7). We used a conjugate gradient ascent algorithm (Nocedal and Wright, 2006) to obtain the joint estimates of $\left\{\theta ,\tau^{2}\right\}$ and executed the algorithm in Python using the JAX software framework (Bradbury et al., 2018) designed for fast computation, compilation, and automatic differentiation. Experiments were run on an internal computing cluster using a 320 NVIDIA P100 Graphical Processing Unit. The maximum likelihood estimates (MLEs) for a were consistently high for data generated from the SGP and low for the UGP data, as expected (Figure 2, middle panel). Additionally, our estimation was able to capture the true values for $\sigma^{2}$ and b (Figure 2, left and right panels).

**Figure 3:**
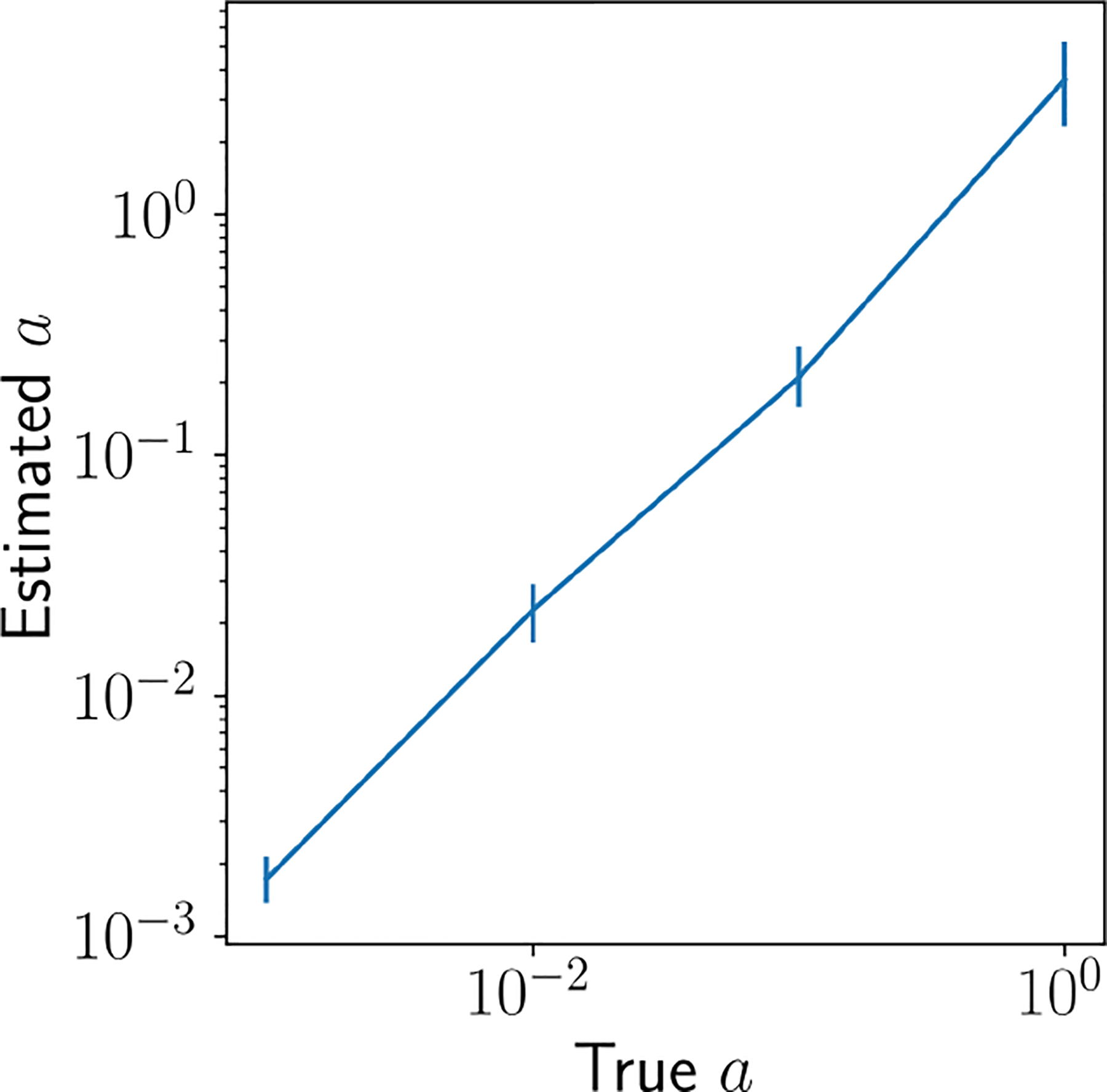
Recovering a with the MGGP MLE. We generated synthetic data from the MGGP at different values for a and subsequently fit the MGGP to these data. We fix all other parameters to their true values. We are able to recover a close approximation of the true value of a.

Next, we generated four data sets from the MGGP for $a\in \left\{10^{-3},10^{-2},10^{-1},10^{0}\right\}$ with sample size fixed at 100. We optimized all parameters jointly by maximizing the marginal multi-group likelihood and examined the estimated value of a for each. We repeated this experiment ten times and found that we could consistently estimate a reasonable value of a (Figure 3). While the estimated values did not exactly coincide with the true values, they showed a desirable monotone relationship. These results reveal that likelihood-based parameter estimation is feasible in the multi-group model and that existing estimation and computational algorithms, such as gradient ascent, can be successfully applied to multi-group models. A formal proof of the consistency of the MLE for a and other kernel parameters, as well as the consistency of the posterior distribution, remains challenging. Consistently estimating GP kernel parameters, even in Euclidean domains, is well-recognized in the literature as a difficult problem (Zhang, 2004; Tang et al., 2021; Li, 2022; Loh and Sun, 2023). This issue remains an important area for future research, as discussed in Section 6.

Turning to the full Bayesian analysis, we generated a simulated data set in the same manner as above for the MGGP described in Section 2. We include group-specific intercepts for the k=2 groups, denoted as $\beta ={\left(\beta_{1},\beta_{2}\right)}^{\text{T}}$, and use $\beta_{1}=1,\beta_{2}=2$ along with group-specific noise variances $\tau_{1}^{2}=0.1,\tau_{2}^{2}=0.3$ to generate the data. We form the $\left(n_{1}+n_{2}\right)\times 2$ binary design matrix F in order to apply the group-specific intercept in the model. For computational efficiency, we fit the collapsed posterior distribution in Equation (3). With $\theta =\left\{a,b,\sigma^{2}\right\}$, the prior distribution in Equation (3) is specified as

(10) $$p\left(\theta ,\left\{\tau_{1}^{2},\tau_{2}^{2}\right\},\beta \right)=IG\left(a|\alpha_{a},\alpha_{a}^{\prime }\right)\times IG\left(b|\alpha_{b},\alpha_{b}^{\prime }\right)\times IG\left(\sigma^{2}|\alpha_{\sigma },\alpha_{\sigma }^{\prime }\right)\;\times \overset{2}{\underset{j=1}{\prod }}IG\left(\tau_{j}^{2}|\alpha_{\tau_{j}},\alpha_{\tau_{j}}^{\prime }\right)\times N\left(\beta |\mu_{\beta },V_{\beta }\right),$$

where we set $\alpha_{a}=\alpha_{a}^{\prime }=\alpha_{b}=\alpha_{b}^{\prime }=\alpha_{\tau_{1}}=\alpha_{\tau_{1}}^{\prime }=\alpha_{\tau_{2}}=\alpha_{\tau_{2}}^{\prime }=5,\alpha_{\sigma }=\alpha_{\sigma }^{\prime }=1,\mu_{\beta }=0$ and $V_{\beta }^{-1}=\text{I}$. We set the values of these parameters for simplicity, but note that, in practice, one may use external information, if available, to elicit prior information. For example, a shrinkage prior on a could be used if there is evidence that the groups are similar.

For inference, we sample from the posterior distribution in Equation (3) using a Hamiltonian No U-Turn Sampling (Hoffman et al., 2014) algorithm as implemented in the Stan programming environment (Stan Development Team, 2020; Riddell et al., 2021). We ran four chains with dispersed initial values for 1, 200 iterations each. Convergence was diagnosed after 200 iterations using visual inspection of autocorrelation plots (Figure 10) and computation of Gelman-Rubin R-hat and Monte Carlo standard errors. The subsequent 4, 000 samples were retained for posterior inference.

**Figure 4:**
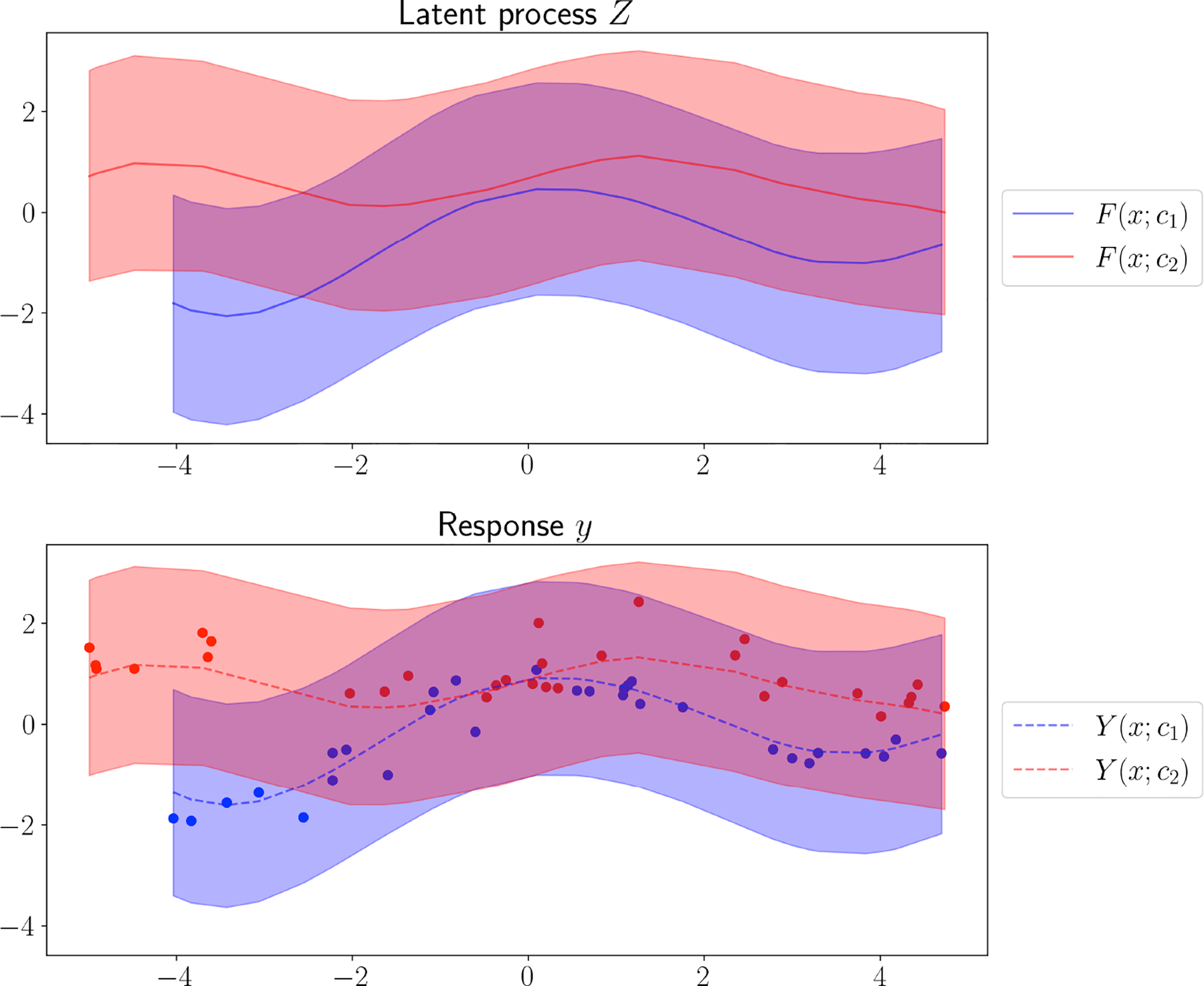
Posterior predictive distribution from the multi-group Gaussian process. The points represent training data; the solid lines show the means of the latent processes F(x;c); the dashed lines represent the predictive means of Y(x;c); and the shaded areas around the lines are twice the standard deviation of the posterior predictive distribution at the corresponding input points.

The posterior median and 95% credible intervals show that the covariance function parameters capture their true values (Figure 11, Table 2). We also sample from the posterior predictive distribution, $p\left(Y\left(x_{0};c_{j}\right)|y,F\right)$ (see Section 2), for a collection of new inputs or test cases (Figure 4). Because all of the MGGP assumptions are encoded in the covariance function, any appropriate method for estimation and inference in standard GPs can be applied.

We next evaluated the MGGP in terms of predicting held-out values in a GP regression task. We generated data from a GP regression model, as in Equation (1), using the SGP, UGP, HGP, and MGGP. We fit these models to each of the data sets using 50% of the data for training, and we test our predictions over the remaining data. We use the predictive mean $\mu^{⋆}=K_{X^{⋆}X}K_{XX}^{-1}y$ as a point prediction for each of the $n^{⋆}$ held-out samples, where $K_{X^{⋆}X}$ is the $n^{⋆}\times n$ matrix of covariance function evaluations for each pair of test and training samples, and $K_{XX}$ is the n×n matrix of covariance function evaluations for each pair of training samples. We center the data for each group around their mean. We compute the mean squared error (MSE) of the predictions, $E=\frac{1}{n^{⋆}}\sum_{i=1}^{n^{⋆}}{\left(y_{i}-\mu_{i}^{⋆}\right)}^{2}$ to evaluate the quality of predictive inference.

**Figure 5:**
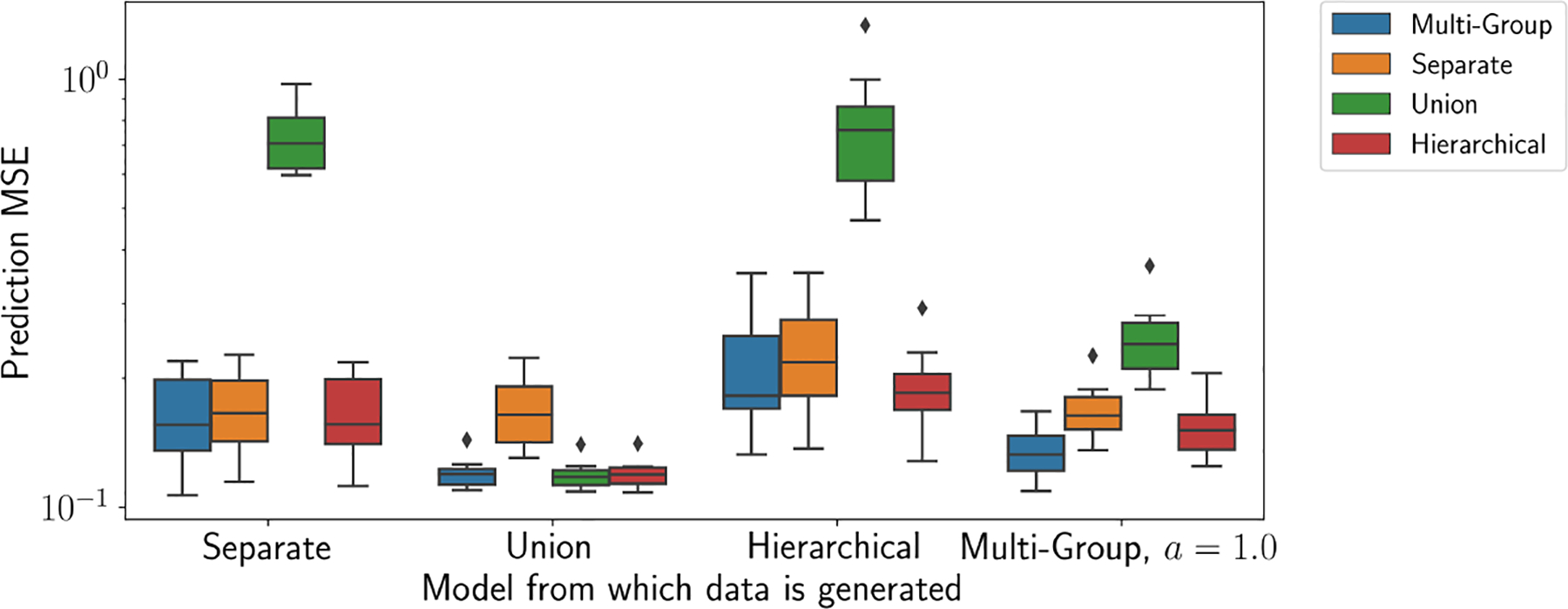
GP predictions with simulated data. We generate data from each of the four models—SGP, UGP, HGP and MGGP—and fit each of these models to all data sets. Prediction error (MSE) was computed on a held-out data set. Figure 9 shows an analogous experiment using the Matérn covariance function.

We find that the MGGP emulates the performance of the SGP, UGP and HGP on their respective simulated data sets (Figure 5). With data generated from the MGGP itself, the MGGP substantially outperforms the other models. While the SGP can be expected to perform well when each group has a large sample size, a primary benefit of MGGPs is their ability to share information across similar groups when the (group) sample size is limited. Thus, we expect MGGPs to excel over SGP when some groups have a small number of samples, but are closely related to other groups.

**Figure 6:**
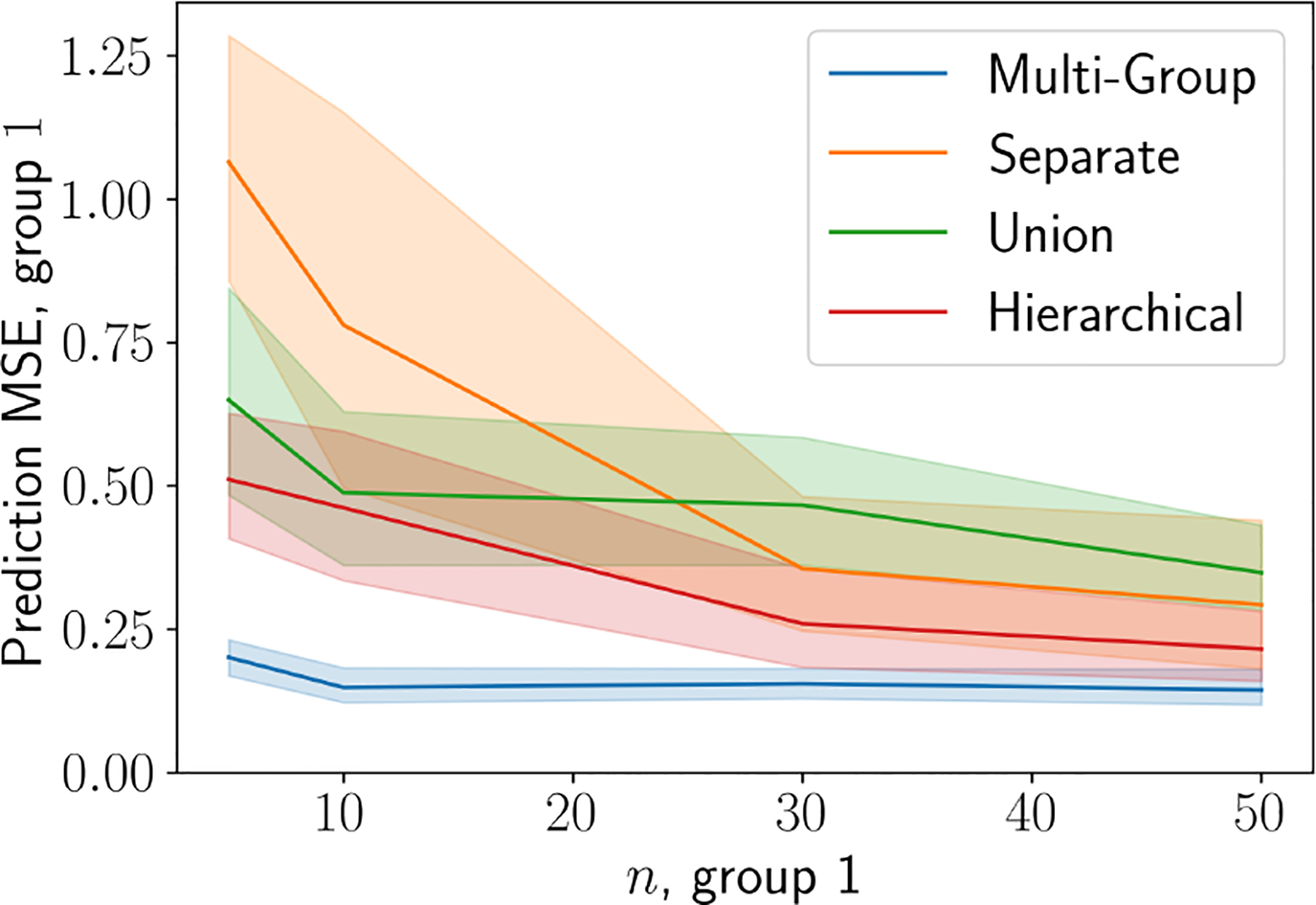
Prediction using simulated data with imbalanced groups. We perform a prediction experiment with k=3 groups. To generate a series of data sets, we fix the sample size of groups $c_{2}$ and $c_{3}$ to be 50, and we vary the sample size of group $c_{1}$.

To test this claim, we conducted another multi-group regression experiment in which we sought to predict the held-out values for one group that contained few samples. Specifically, we generated synthetic data consisting of three groups, where group 1 and group 2 are similar to one another, and group 3 is dissimilar from the other two. We then generate a series of data sets, with sample size of group 2 and 3 being 50 and varying the number of samples in group 1 to take values in {5, 10, 30, 50}. Then, we fit the SGP, UGP, HGP and MGGP to each of the data sets, using 50% of the data for training, and testing predictions on the other 50%. We find that the MGGP model outperforms the other methods, especially when the sample size for group 1 is small (Figure 6). This result shows that the MGGP appears to thrive when the sample size for some groups is limited, as it most effectively leverages information from similar groups while acknowledging the group structure.

### 4.3 Partially observed coordinates

A key benefit of our process-based modeling framework is its versatility in dealing with situations where we have partially observed coordinates. This setting arises in situations where not all group labels $c_{j}\in 𝒞$ have yielded measurements on an identical set of $x_{j}\in 𝒳$, which leads to an imbalance. Despite this, inference still proceeds seamlessly using the framework described in Section 2. In fact, the simulated data in our experiments reflect this exact setup: the $x_{j}$s within each group rarely overlapped across groups, meaning that almost all $x_{j}$s were exclusive to a single group. For example, the observed measurements for two groups in our previous example have minimal overlap (Figure 4), and the MSE values (Figure 5) were computed under this disjoint groups of $x_{j}$s setup.

## 5. Application to GTEx tissue samples

We applied the MGGP to a large gene expression data set collected by the Genotype-Tissue Expression (GTEx) project (Consortium et al., 2020). The GTEx v8 data contain measurements from 17,382 samples that span 52 tissue types collected from 838 human donors; see Appendix K for a full list of tissue types and the sample size for each tissue. Along with gene expression profiling, a variety of additional metadata characteristics are collected, including demographic variables and tissue health measurements.

In these experiments, we use GP regression models to analyze the relationship between a sample’s gene expression profile and its *ischemic time*–the duration of time between death and tissue collection. Previous work has shown a robust relationship between gene expression and ischemic time (Musella et al., 2013; Ferreira et al., 2018); however, whether this relationship exhibits tissue-specific patterns remains largely untested. In these experiments, the groups correspond to tissue of origin for each sample.

As an initial test with the GTEx data, we applied the MGGP to samples from just two tissue types at a time. These experiments aim to validate that MGGP regression can appropriately model known associations across similar groups and estimate pairwise similarity between groups.

In a preliminary experiment, we examined three tissue types: anterior cingulate cortex (n=172), frontal cortex (n=200), and coronary artery (n=238). First, for each of the three pairs of tissues, we fit the MGGP model with MLEs, as described in Section 4.2, using the multi-group RBF covariance function (Equation (7)). In this experiment, we fixed a to one value in a preset range, and found the MLEs of the remaining parameters. This experiment aims to justify our interpretation of a using a real data set where we know the similarity between certain groups (i.e., tissues). In practice, a is estimated using an MLE in all other simulations and applications. Using these MLEs and the fixed a, we then computed the log marginal likelihood of the data, $\text{log}\;p\left(y|X,a,\hat{b},\hat{\sigma^{2}},\hat{\tau^{2}}\right)=-\frac{k}{2}2\pi -\frac{1}{2}\text{det}\left(K_{XX}+\hat{\tau^{2}}I\right)-\frac{1}{2}y^{\text{T}}{\left(K_{XX}+\hat{\tau^{2}}I\right)}^{-1}y$, where $K_{XX}$ is the $\left(n_{1}+n_{2}\right)\times \left(n_{1}+n_{2}\right)$ matrix of covariance function evaluations for each pair of samples. We also fit the SGP and UGP for each pair of tissues using the standard RBF, and computed the log marginal likelihood of the data under these models.

**Figure 7:**
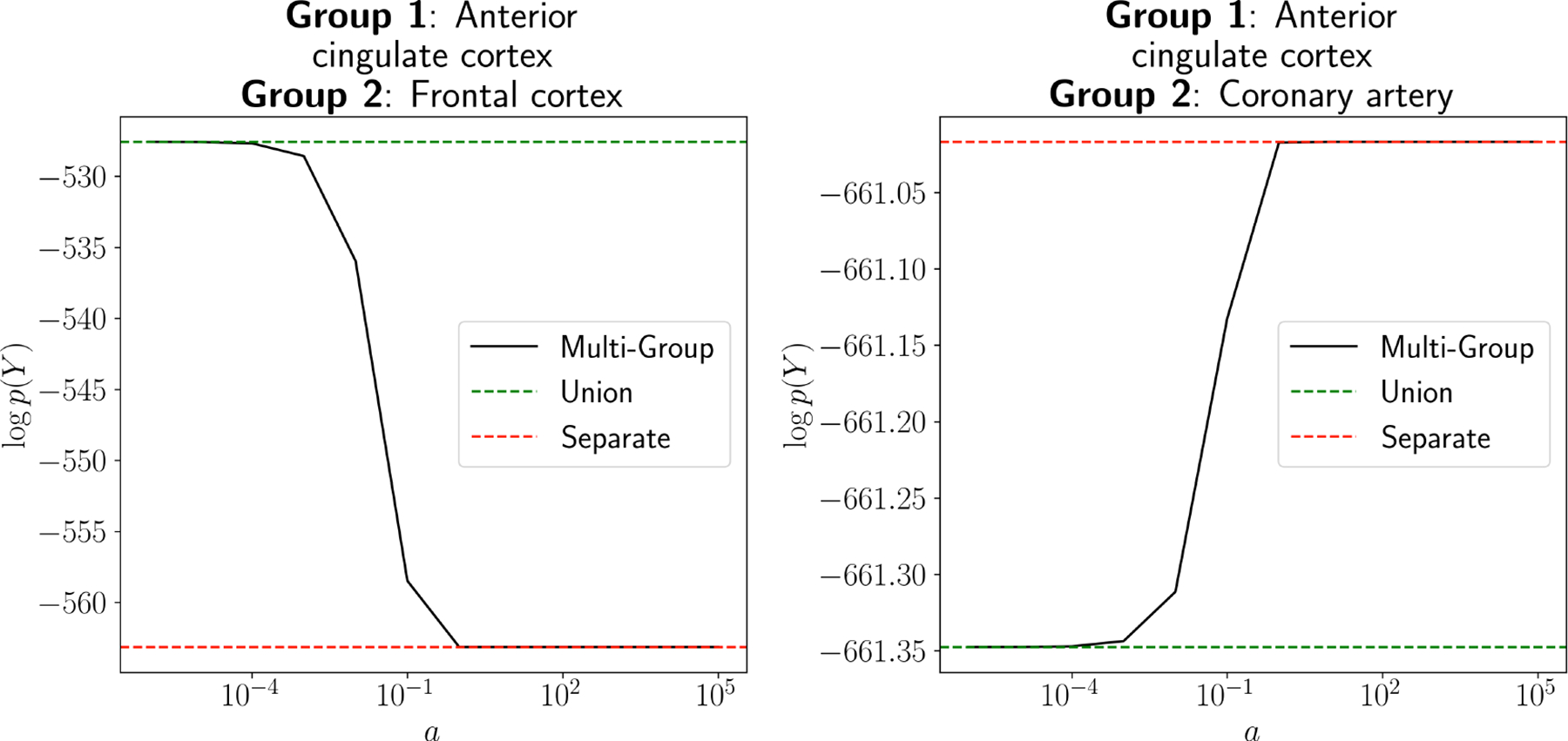
Likelihood of GTEx gene expression data under the MGGP model. For each pair of tissues, we computed the log marginal likelihood of the data under the MGGP model with a set to be a range of values. Similar tissue types (e.g., anterior cingulate cortex and frontal cortex) prefer low values of a, while more dissimilar tissues (e.g., anterior cingulate cortex and coronary artery) prefer high values of a.

Examining the log marginal likelihood across varying values of a (Figure 7), we found that two brain tissue types that are expected to be similar to one another—anterior cingulate and frontal cortex—showed a higher marginal likelihood under small values of (a⪅0.01), while tissues that have unique expression patterns—anterior cingulate cortex and coronary artery—showed a higher marginal likelihood under large values of a(a⪆10). In both cases, the MGGP gracefully recovered the Separate and Union marginal likelihoods for a→∞ and a→0, respectively. This result implies that MGGP is a viable strategy not only for sharing information across groups, but also for quantifying the group relationships themselves.

**Figure 8:**
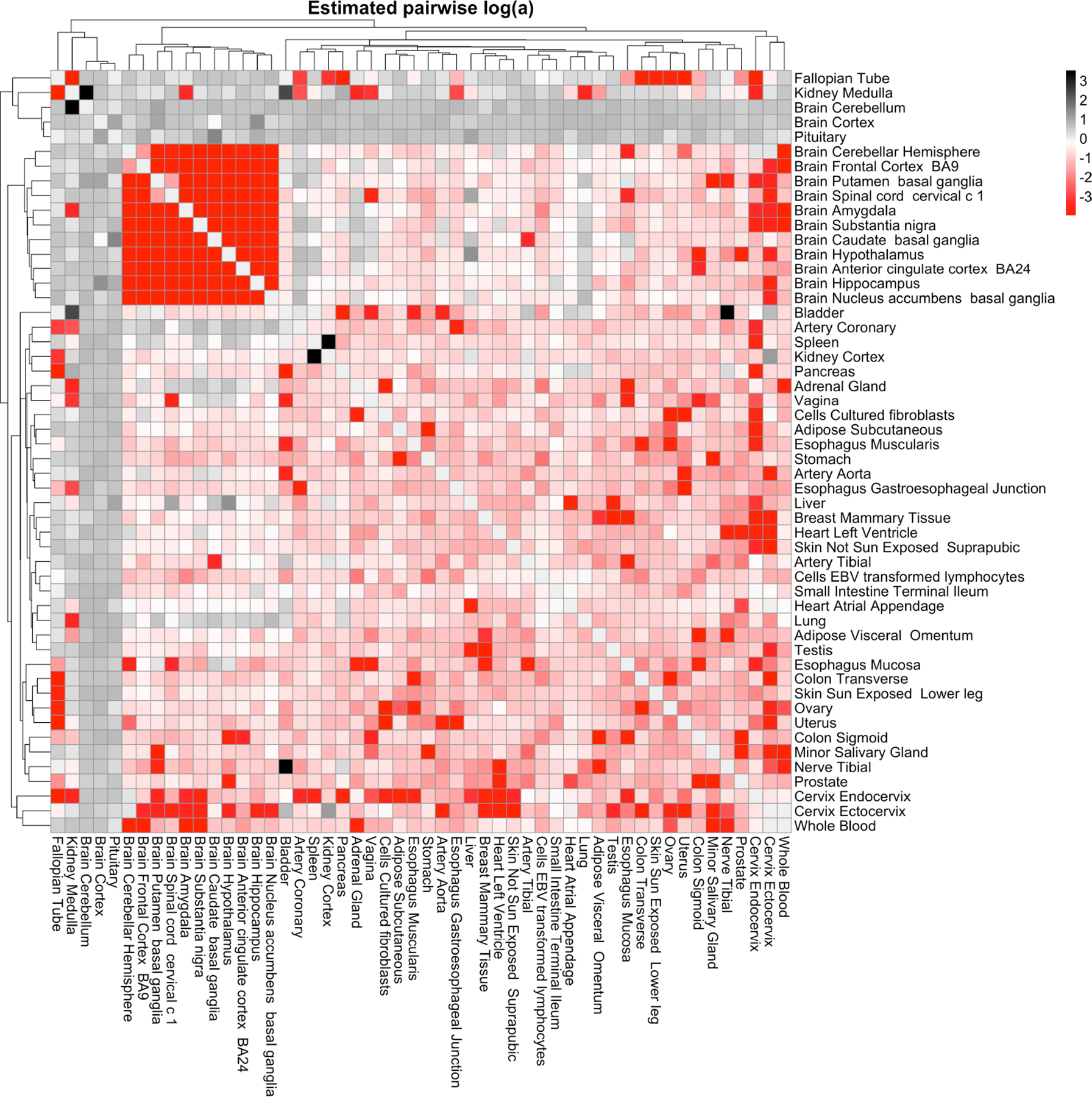
Estimation of a for each pair of GTEx tissue types. Cell ij in the heatmap represents ${\text{log}}_{10}\left(a_{ij}\right)$, where $a_{ij}$ is the MLE of a when fitting the MGGP model using tissues i and j. Lower values of a (red) indicate higher similarity, while higher values of a (black) indicate lower similarity.

We also conduct a similar experiment where we obtain MLEs of a (along with all other model and covariance parameters) from the data. Here, we apply the model to all 52 tissue types. We fit the MGGP for every pair of tissues, and extract ${\hat{a}}_{MLE}$ for each pair. This experiment yields $\frac{1}{2}(52\times 51)=1326$ estimated values of a (one for each pair of tissue types). The estimated values of a reflect many of the expected relationships between the tissue types (Figure 8). Notably, we report 11 regions of the brain yielding low values for a, which suggests that gene expression in these tissue types changes in a similar manner as ischemic time changes.

Finally, we conduct a fully Bayesian analysis of the GTEx data using the MGGP model. Here, we analyze the 11 brain tissue types, which comprise a total of 2218 samples, using Equation 3. For ease of visualization and demonstration, we use the expression of just one gene, *TXNIP*, as our explanatory variable, and use each sample’s ischemic time as the response as before. We use the same modeling approach as in our simulation study (Section 4.2), adapting the model for 11 groups. Again, we fit the collapsed likelihood in Equation 3 incorporating group-specific intercepts in F and group-specific variances in $D_{\tau }$. We run four chains with dispersed initial values for 300 iterations each. Convergence is diagnosed after 100 iterations using visual inspection of autocorrelation plots. The subsequent 800 samples are retained for posterior inference.

Using posterior summaries of the covariance function parameters, intercepts, and noise variances (Table 3), we find that the MGGP successfully models these relationships across groups. Moreover, the predictive processes demonstrate the similarities and differences between the groups (Supplementary Figures 13, 14). For example, we see that while all brain regions tend to exhibit a similar relationship between gene expression levels and ischemic time, this relationship shows a distinct trend in the cerebellum and putamen. We find that the MGGP predictive processes capture these subtle group relationship differences.

## 6. Discussion

We develop multi-group Gaussian process (MGGP) models as a flexible approach for modeling complex dependencies in data sets with subgroup structure. We present several options for constructing valid covariance functions on $R^{p}\times 𝒞$, and we show that this structure generalizes existing GP models. We emphasize that the MGGP novelty is in the construction of the covariance functions, enabling all GP inference strategies applicable to MGGPs. We demonstrate the behavior of the MGGP through several simulation experiments and an application to gene expression data with ischemic time measurements for 52 distinct tissues.

Several future directions remain to be explored. First, this paper lays the groundwork for developing new positive definite covariance functions on $R^{p}\times 𝒞$. An interesting direction is to construct covariance functions whose within-group and between-group correlations exhibit fundamentally different structure (e.g., the within-group correlation may be Matérn families, while the between group correlation may be RBF families). Second, as briefly mentioned in Section 2, recent advances in classes of GPs that scale learning to massive data sets can be applied to the MGGP. For example, sparsity-inducing GPs have received much attention recently (see, e.g., Datta et al., 2016; Katzfuss and Guinness, 2021; Peruzzi et al., 2022), and such methods can be applied to the class of multi-group models presented here. Third, a linear, yet nonseparable, MGGP kernel remains to be explored: A naive approach is to assign different linear coefficients to different groups; however, this leads to a separable kernel.Fourth, there are opportunities to explore alternate GP representations by adopting Mercer’s theorem, i.e., an eigenfunction-based decomposition, to help build new covariance functions. The main challenge here is to identify the suitable eigenfunctions with both continuous and categorical components. Fifth, the consistency of MLEs and the posterior consistency of kernel parameters remain open and challenging problems. Sixth, the multi-group kernels can serve as valid cross-covariance functions for multivariate spatial processes by treating the categories as indices for the elements of a vector process. They present sparser parametric forms than cross-covariance functions resulting from the widely employed linear models of coregionalization in spatial statistics (see, e.g., Gelfand et al., 2004; Banerjee and Johnson, 2006; Guhaniyogi et al., 2013, with applications in agronomy, ecology and environmental sciences) and can also serve as alternatives in process-based factor models (Zhang and Banerjee, 2022; Davies et al., 2022) and as candidates to build highly multivariate graphical GPs (Dey et al., 2022). Finally, given that the MGGPs are well-defined stochastic processes, they can be introduced in any process-based model, perhaps replacing more customary choices that do not allow both continuous and categorical variables simultaneously. Thus, there could be benefits from using a multi-group process in classification, latent variable models, and other model types. We envision the MGGP being a flexible tool in diverse contexts.

## Supplementary Material

1

## Figures and Tables

**Table 1:** **Candidate functions for completely monotone functions** ϕ **and positive functions with completely monotone derivatives** ψ. Here, a,c,ν>0,b>1,0<α,β,γ≤1.

ϕ(t)	ψ(t)
$\text{exp}\left(-ct^{\gamma }\right)$	${\left(at^{\alpha }+1\right)}^{\beta }$
${\left(2^{\nu -1}\Gamma (\nu )\right)}^{-1}{\left(ct^{1/2}\right)}^{\nu }K_{\nu }\left(ct^{1/2}\right)$	$\text{log}\left(at^{\alpha }+b\right)/\text{log}b$
${\left(1+ct^{\gamma }\right)}^{-\nu }$	$\left(at^{\alpha }+\beta \right)/\left(\beta \left(at^{\alpha }+1\right)\right)$
$2^{\nu }{\left(\text{exp}\left(ct^{1/2}\right)+\text{exp}\left(-ct^{1/2}\right)\right)}^{-\nu }$	

**Table 2:** Parameter posterior summaries for simulated Bayesian analysis. Posterior summaries are presented as 50(2.5; 97.5) percentiles in the third column.

Parameter	True	Posterior percentiles
a	1.0	1.66 (0.82; 4.29)
b	1.0	0.47 (0.29; 0.88)
$\sigma^{2}$	1.0	1.41 (0.76; 2.98)
$\tau_{1}$	0.1	0.14 (0.09; 0.21)
$\tau_{2}$	0.3	0.29 (0.2; 0.47)
$\beta_{1}$	1.0	0.73 (−0.38; 1.66)
$\beta_{2}$	2.0	1.86 (0.77; 2.8)

**Table 3:** Parameter posterior summaries for Bayesian analysis of GTEx data. Posterior summaries are presented as 50(2.5; 97.5) percentiles in the second column. Subscripts on parameter names indicate group labels.

Parameter	Posterior percentiles	Parameter	Posterior percentiles
$\sigma^{2}$	0.49 (0.28; 0.82)	$\beta_{11}$	−0.02 (−0.48; 0.67)
b	0.63 (0.32; 1.16)	$\tau_{1}^{2}$	0.79 (0.63; 1.0)
a	0.45 (0.28; 0.93)	$\tau_{2}^{2}$	0.97 (0.81; 1.14)
$\beta_{1}$	−0.04 (−0.51; 0.56)	$\tau_{3}^{2}$	0.89 (0.75; 1.12)
$\beta_{2}$	−0.08 (−0.58; 0.47)	$\tau_{4}^{2}$	0.86 (0.74; 1.07)
$\beta_{3}$	0.01 (−0.46; 0.61)	$\tau_{5}^{2}$	0.87 (0.75; 1.01)
$\beta_{4}$	0.64 (0.07; 1.17)	$\tau_{6}^{2}$	0.89 (0.74; 1.09)
$\beta_{5}$	0.7 (0.12; 1.28)	$\tau_{7}^{2}$	0.85 (0.68; 1.05)
$\beta_{6}$	−0.0 (−0.46; 0.59)	$\tau_{8}^{2}$	0.86 (0.74; 1.01)
$\beta_{7}$	−0.04 (−0.46; 0.52)	$\tau_{9}^{2}$	0.8 (0.67; 1.0)
$\beta_{8}$	−0.1 (−0.48; 0.46)	$\tau_{10}^{2}$	0.89 (0.67; 1.09)
$\beta_{9}$	0.18 (−0.3; 0.68)	$\tau_{11}^{2}$	0.88 (0.69; 1.14)
$\beta_{10}$	−0.03 (−0.47; 0.51)		

## Appendix A. Proof of Proposition 2

Proof

First, assume $K_{2}$ is positive definite, and let the observation set be $\left\{c_{1},\cdots ,c_{k}\right\}$. Then the covariance matrix is exactly C, which is positive definite as well.

Then assume C is positive definite, and define X be a random variable that follows a k-dimension Gaussian distribution: X~N(0,C). Given observations $a_{1},\cdots ,a_{n}\subset \left\{c_{1},\cdots ,c_{k}\right\}$, let Y be a n-dimensional random variable such that $Y_{i}=X_{a_{i}}$, then $K\left(a_{i},a_{j}\right)=C_{a_{i},a_{j}}=\text{Cov}\left(X_{a_{i}},X_{a_{j}}\right)=\text{Cov}\left(Y_{i},Y_{j}\right)$. That is, K is positive definite.

■

## Appendix B. Proof of Corollary 4

Proof By Proposition 2, $K_{2}$ is positive definite if and only if C is positive definite. Under the assumption of homogeneity, $C=\left[\begin{array}{cccc} 1 & b & \cdots & b\\ b & 1 & \cdots & b\\ \vdots & \vdots & \ddots & \vdots \\ b & b & \cdots & 1 \end{array}\right]$. We can rewrite $C=(1-b){\text{I}}_{k}+b1_{k\times k}$. Recall that $1_{k\times k}$ has eigenvalue p with multiplicity 1, and the corresponding eigenvector is $1_{k}$. Moreover, 0 is another eigenvalue with multiplicity k-1. As a result, eigenvalues of Σ are 1-b+bk with multiplicity 1 and 1-b with multiplicity −1. We can conclude that C is positive definite if and only if $-\frac{1}{k-1}\leq b\leq 1$. ■

## Appendix C. Proof of Theorem 6

Restricting the GP to a subset of the original domain is again a GP (Rasmussen and Williams, 2005). Hence, if we can isometrically embed 𝒞 to an Euclidean space $R^{p^{\prime }}$ by some mapping $\iota :𝒞\to R^{p^{\prime }}$ such that $d_{ij}≔d\left(c_{i},c_{j}\right)=\left\|\iota \left(c_{i}\right)-\iota \left(c_{j}\right)\right\|$, then an isotropic GP on $R^{p^{\prime }}$ induces a GP on the image ι(𝒞). To construct valid covariance functions, the explicit form of ι is not necessary. As long as such embedding exists, we can construct semi-isotropic covariance functions.

**Lemma 13** (Maehara (2013)) *Let* $G_{ij}=\frac{1}{2}\left(d\left(c_{1},c_{i}\right)+d\left(c_{1},c_{j}\right)-d\left(c_{i},c_{j}\right)\right)$ *be the* k×k *Gram matrix of* d. *Then there exists an isotropic embedding* $\iota :𝒞\to R^{p^{\prime }}$ *if and only if* G *is positive semi-definite with rank at most* $p^{\prime }$.

**Proof** By the isometric embedding, finding K is equivalent to finding a semi-isotropic covariance function $K_{0}$ in $R^{p}\times R^{p^{\prime }}$. For any completely monotone function $\varphi :R_{+}\to R$ and positive function $\psi :R_{+}\to R_{+}$ with a completely monotone derivative, Gneiting (2002) proved that $K_{0}\left(\left(x_{1},x_{1}^{\prime }\right),\left(x_{2},x_{2}^{\prime }\right)\right)=\frac{\sigma^{2}}{{\left(\psi \left({\left\|x_{1}^{\prime }-x_{2}^{\prime }\right\|}^{2}\right)\right)}^{p/2}}\varphi \left(\frac{{\left\|x_{1}-x_{2}\right\|}^{2}}{\psi \left({\left\|x_{1}^{\prime }-x_{2}^{\prime }\right\|}^{2}\right)}\right)$ is positive definite for any $\sigma^{2}>0$. As a result, $K\left(\left(x,c_{i}\right),\left(x^{\prime },c_{j}\right)\right)≔K_{0}\left(\left(x,\iota \left(c_{i}\right)\right),\left(x^{\prime },\iota \left(c_{j}\right)\right)\right)$ is positive definite. ■

## Appendix D. Proof of Theorem 7

Recall the general form of Bochner’s Theorem for a locally compact Abelian group:

**Lemma 14 (Bochner’s Theorem)** *Let* G *be a locally compact Abelian group and* $\hat{G}$ *be its dual group, then for any continuous positive-definite function* K *on* G, *there exists a unique positive measure* μ *on* $\hat{G}$ *such that*

$$K\left(g\right)=\int_{\hat{G}}\xi \left(g\right)d\mu \left(\xi \right).$$

Note that $G=R^{p}\times Z_{2}$ is a locally compact Abelian group and the dual group is $\hat{G}=R^{p}\times U_{2}$, where $U_{2}$ is the group of second roots of unity, that is, $U_{2}=\{1,-1\}.\hat{G}$ acts on G as

$$(\omega ,z)((x,l))=e^{-2\pi i\omega x}z^{l}.$$

The spectral measure of K, denoted by μ on $R^{p}\times U_{2}$ splits as $\mu_{1}\times \mu_{2}$ where $\mu_{1}$ is a measure on $R^{p}$ and $\mu_{2}$ is a measure on $U_{2}$. Then we claim that

$$K\left(x,l\right)=\sum_{z\in U_{2}}\int_{R^{p}}e^{-2\pi i\omega x}z^{l}\rho \left(\omega ,z\right)d\omega ,$$

where $\rho (\omega ,1)=\frac{1}{2}\rho_{w}(\omega )+\frac{1}{2}\rho_{c}(\omega )$ and $\rho (\omega ,-1)=\frac{1}{2}\rho_{w}(\omega )-\frac{1}{2}\rho_{c}(\omega )$. We derive the left hand side from the right hand side. First consider the case when l=0:

$$\sum_{z\in U_{2}}\int_{R^{p}}e^{-2\pi i\omega x}z^{0}\rho (\omega ,z)d\omega \;=\frac{1}{2}\int_{R^{p}}e^{-2\pi i\omega x}\left(\rho_{w}(\omega )+\rho_{c}(\omega )\right)d\omega +\frac{1}{2}\int_{R^{p}}e^{-2\pi i\omega x}\left(\rho_{w}(\omega )-\rho_{c}(\omega )\right)d\omega \;=\int_{R^{p}}e^{-2\pi i\omega x}\rho_{w}(\omega )d\omega \;=K_{w}\left(x\right)=K\left(x,0\right).$$

Similarly, when l=1,

$$\sum_{z\in U_{2}}\int_{R^{p}}e^{-2\pi i\omega x}z^{1}\rho (\omega ,z)d\omega \;=\frac{1}{2}\int_{R^{p}}e^{-2\pi i\omega x}\left(\rho_{w}(\omega )+\rho_{c}(\omega )\right)d\omega -\frac{1}{2}\int_{R^{p}}e^{-2\pi i\omega x}\left(\rho_{w}(\omega )-\rho_{c}(\omega )\right)d\omega \;=\int_{R^{p}}e^{-2\pi i\omega x}\rho_{c}(\omega )d\omega \;=K_{c}\left(x\right)=K\left(x,1\right).$$

As a result, K is positive definite ⟺ρ is a positive measure $⟺\rho_{w}\geq \rho_{c}$, and the Theorem follows.

## Appendix E. Proof of Theorem 9

The generalized spectral measure of K, denoted by μ (with density ρ) on $R^{p}\times U_{2}\times U_{2}$, splits as $\mu_{1}\times \mu_{2}$ where $\mu_{1}$ is a measure on $R^{p}$ and $\mu_{2}$ is a positive semi-definite measure on $U_{2}\times U_{2}$. Then

$$K\left(x,l,l^{\prime }\right)=\sum_{z\in U_{2}}\sum_{z^{\prime }\in U_{2}}\int_{R^{p}}e^{-2\pi i\omega x}z^{l}\bar{z^{\prime l^{\prime }}}\rho \left(\omega ,z,z^{\prime }\right)d\omega .$$

$$\text{We}\;\text{claim}\;\text{that}\;\rho \left(\omega ,z,z^{\prime }\right)=\left\{\begin{array}{ll} \frac{1}{4}\left(\rho_{0}(\omega )+2\rho_{c}(\omega )+\rho_{0}(\omega )\right) & z=z^{\prime }=1\\ \frac{1}{4}\left(\rho_{0}(\omega )-\rho_{1}(\omega )\right) & zz^{\prime }=-1\\ \frac{1}{4}\left(\rho_{0}(\omega )-2\rho_{c}(\omega )+\rho_{1}(\omega )\right) & z=z^{\prime }=-1. \end{array}\right..$$

We work with the right hand side. First consider the case when $l=l^{\prime }=0$:

$$\sum_{z\in U_{2}}\sum_{z^{\prime }\in U_{2}}\int_{R^{p}}e^{-2\pi i\omega x}z^{0}\bar{z^{\prime 0}}\rho \left(\omega ,z,z^{\prime }\right)d\omega \;=\frac{1}{4}\int_{R^{p}}e^{-2\pi i\omega x}\left\{\rho_{0}(\omega )+2\rho_{c}(\omega )+\rho_{1}(\omega )\right.\;\left.\;+\rho_{0}(\omega )-\rho_{1}(\omega )+\rho_{0}(\omega )-\rho_{1}(\omega )+\rho_{0}(\omega )-2\rho_{c}(\omega )+\rho_{1}(\omega )\right\}d\omega \;=\int_{R^{p}}e^{-2\pi i\omega x}\rho_{0}\left(\omega \right)d\omega =K_{0}\left(x\right)=K\left(x,0,0\right).$$

Similarly, when $l=0,l^{\prime }=1$,

$$\sum_{z\in U_{2}}\sum_{z^{\prime }\in U_{2}}\int_{R^{p}}e^{-2\pi i\omega x}z^{0}\bar{z^{\prime 1}}\rho \left(\omega ,z,z^{\prime }\right)d\omega \;=\frac{1}{4}\int_{R^{p}}e^{-2\pi i\omega x}\left(\rho_{0}(\omega )+2\rho_{c}(\omega )+\rho_{1}(\omega )\right.\;\left.\;-\rho_{0}(\omega )+\rho_{1}(\omega )+\rho_{0}(\omega )-\rho_{1}(\omega )-\rho_{0}(\omega )+2\rho_{c}(\omega )-\rho_{1}(\omega )\right)d\omega \;=\int_{R^{p}}e^{-2\pi i\omega x}\rho_{c}\left(\omega \right)d\omega =K_{c}\left(x\right)=K\left(x,0,1\right).$$

When $l=l^{\prime }=1$,

$$\sum_{z\in U_{2}}\sum_{z^{\prime }\in U_{2}}\int_{R^{p}}e^{-2\pi i\omega x}z^{1}\bar{z^{\prime 1}}\rho \left(\omega ,z,z^{\prime }\right)d\omega \;=\frac{1}{4}\int_{R^{p}}e^{-2\pi i\omega x}\left(\rho_{0}(\omega )+2\rho_{c}(\omega )+\rho_{1}(\omega )-\rho_{0}(\omega )+\rho_{1}(\omega )-\rho_{0}(\omega )\right.\;\left.\;+\rho_{1}(\omega )+\rho_{0}(\omega )-2\rho_{c}(\omega )+\rho_{1}(\omega )\right)d\omega \;=\int_{R^{p}}e^{-2\pi i\omega x}\rho_{1}\left(\omega \right)d\omega =K_{1}\left(x\right)=K\left(x,1,1\right).$$

As a result, K is positive definite ⟺ρ is a positive semi-definite measure $⟺\left(\rho_{0}+2\rho_{c}+\right.\left.\rho_{1}\right)\left(\rho_{0}-2\rho_{c}+\rho_{1}\right)-{\left(\rho_{0}-\rho_{1}\right)}^{2}=4\left(\rho_{0}\rho_{1}-\rho_{c}^{2}\right)\geq 0$, and the Theorem follows.

## Appendix F. Proof of Theorem 10

**Proof** Given a Gaussian random field Z on 𝒴×𝒞 with covariance function K, we prove that $\tilde{Z}≔\Phi (Z)$ is a Gaussian k-variate random field on 𝒴 with cross-covariance function $\tilde{K}$. It suffices to check that $\text{Cov}\left(\tilde{Z}(x),\tilde{Z}\left(x^{\prime }\right)\right)=\tilde{K}\left(x,x^{\prime }\right)$, for any $x,x^{\prime }\in 𝒴$.

$$\text{Cov}\left(\tilde{Z}(x),\tilde{Z}\left(x^{\prime }\right)\right)=\text{Cov}\left({\left[Z\left(x,c_{1}\right),\cdots ,Z\left(x,c_{k}\right)\right]}^{\text{T}},{\left[Z\left(x^{\prime },c_{1}\right),\cdots ,Z\left(x^{\prime },c_{k}\right)\right]}^{\text{T}}\right)\;=\left[\begin{array}{cccc} K\left(\left(x,c_{1}\right),\left(x^{\prime },c_{1}\right)\right) & K\left(\left(x,c_{1}\right),\left(x^{\prime },c_{2}\right)\right) & \cdots & K\left(\left(x,c_{1}\right),\left(x^{\prime },c_{k}\right)\right)\\ K\left(\left(x,c_{2}\right),\left(x^{\prime },c_{1}\right)\right) & K\left(\left(x,c_{2}\right),\left(x^{\prime },c_{2}\right)\right) & \cdots & K\left(\left(x,c_{2}\right),\left(x^{\prime },c_{k}\right)\right)\\ \vdots & \vdots & \ddots & \vdots \\ K\left(\left(x,c_{k}\right),\left(x^{\prime },c_{1}\right)\right) & K\left(\left(x,c_{k}\right),\left(x^{\prime },c_{2}\right)\right) & \cdots & K\left(\left(x,c_{k}\right),\left(x^{\prime },c_{k}\right)\right) \end{array}\right]\;=\left[\begin{array}{cccc} {\tilde{K}}_{11} & {\tilde{K}}_{12} & \cdots & {\tilde{K}}_{1k}\\ {\tilde{K}}_{21} & {\tilde{K}}_{22} & \cdots & {\tilde{K}}_{2k}\\ \vdots & \vdots & \ddots & \vdots \\ {\tilde{K}}_{k1} & {\tilde{K}}_{k2} & \cdots & {\tilde{K}}_{kk} \end{array}\right]\;=\tilde{K}\left(x,x^{\prime }\right).$$

Then assume $\tilde{Z}$ is a Gaussian k-variate random field on 𝒴. We prove that $Z:\Phi^{-1}(\tilde{Z})$ is a Gaussian random field on 𝒴×𝒞 with covariance function K. It suffices to check that $\text{Cov}\left(Z\left(x,c_{i}\right),Z\left(x^{\prime },c_{j}\right)\right)=K\left(\left(x,c_{i}\right),\left(x^{\prime },c_{j}\right)\right)$ for any x,y∈𝒴,i,j=1,⋯,k. Then,

$$\text{Cov}\left(Z\left(x,c_{i}\right),Z\left(x^{\prime },c_{j}\right)\right)\left.\left.=\text{Cov}\left({\tilde{Z}}_{i}(x)\right),{\tilde{Z}}_{i}\left(x^{\prime }\right)\right)\right)\;=\tilde{K}{\left(x,x^{\prime }\right)}_{ij}\;=K\left(\left(x,c_{i}\right),\left(x^{\prime },c_{j}\right)\right).$$

■

## Appendix G. Proof of Theorem 12

Similar to the proof of Theorem 6, it suffices to construct a covariance function on $R^{p}\times R^{p^{\prime }}\times R^{p^{\prime \prime }}$. Then the Theorem follows Proposition 1 in Apanasovich and Genton (2010) naturally.

## Appendix H. More covariance functions

We provide more semi-isotropic covariance functions below from Cressie and Huang (1999) derived from spectral densities.

(11)
$$K\left(\left(x,c_{i}\right),\left(x^{\prime },c_{j}\right)\right)=\frac{\sigma^{2}}{{\left(ad_{ij}+1\right)}^{p/2}}\text{exp}\left\{-\frac{b^{2}{\left\|x-x^{\prime }\right\|}^{2}}{ad_{ij}+1}\right\},$$

(12)
$$K\left(\left(x,c_{i}\right),\left(x^{\prime },c_{j}\right)\right)=\frac{\sigma^{2}\left(a^{2}d_{ij}^{2}+1\right)}{{\left[{\left(a^{2}d_{ij}^{2}+1\right)}^{2}+b^{2}{\left\|x-x^{\prime }\right\|}^{2}\right]}^{\frac{p+1}{2}}},$$

(13)
$$K\left(\left(x,c_{i}\right),\left(x^{\prime },c_{j}\right)\right)=\frac{\sigma^{2}\left(ad_{ij}+1\right)}{{\left[{\left(ad_{ij}+1\right)}^{2}+b^{2}{\left\|x-x^{\prime }\right\|}^{2}\right]}^{\frac{p+1}{2}}},$$

(14)
$$K\left(\left(x,c_{i}\right),\left(x^{\prime },c_{j}\right)\right)=\sigma^{2}\text{exp}\left\{-a^{2}d_{ij}^{2}-b^{2}{\left\|x-x^{\prime }\right\|}^{2}-cd_{ij}^{2}{\left\|x-x^{\prime }\right\|}^{2}\right\},$$

(15)
$$K\left(\left(x,c_{i}\right),\left(x^{\prime },c_{j}\right)\right)=\sigma^{2}\text{exp}\left\{-ad_{ij}-b^{2}{\left\|x-x^{\prime }\right\|}^{2}-cd_{ij}{\left\|x-x^{\prime }\right\|}^{2}\right\}.$$

**Table 4:** Summary of constructions of kernel (7–9), (11–15).

Kernel	Class	Details
(7)	Gneiting	$\phi (t)=e^{bt^{1/2}},\psi (t)=a^{2}t+1$
(8)	Gneiting	$\phi (t)=\frac{{\left(bt^{1/2}/\sqrt{c}\right)}^{\nu }}{2^{\nu -1}\Gamma (\nu )}K_{\nu }\left(bt^{1/2}\sqrt{c}\right),\psi (t)=\frac{a^{2}t+c}{c\left(a^{2}t+1\right)}$
(9)	Gneiting	Setν=1/2 in (8)
(11)	Cressie & Huang	Example 2
(12)	Cressie & Huang	Example 3
(13)	Cressie & Huang	Example 4
(14)	Cressie & Huang	Example 5
(15)	Cressie & Huang	Example 6

Table 4 summaries the kernel constructions:

## Appendix I. Extension to unknown distance $d_{ij}$

In the situation where $d_{ij}$ is unknown, we instead parameters the kernels discussed in main sections (Equations (7–9), (11–15)) as $a_{ij}≔ad_{ij}$, so that the parameters are $\left(\sigma^{2},b,A\right)$ where $A=\left(a_{ij}\right)$ is a k by k matrix. The constraints on A are from the metric axioms:

$$A_{ii}=0,\;A_{ij}=A_{ji},\;A_{ij}+A_{jl}\geq A_{il},\forall i,j,l=1,\cdots ,k.$$

The MLE is then over the new parameter space, under the above constraints. Fortunately, these constraints are all linear, so optimization methods such as L-BFGS can still be applied. In this case, the parameter $a_{ij}$ is purely data-driven, and can be interpreted as the dissimilarity between group i and group j, as desired.

## Appendix J. Code

Our GitHub repository is https://github.com/andrewcharlesjones/multi-group-GP. This repository contains downloadable code for the models and experiments to reproduce the analysis in the paper. We provide a Python package for model fitting, computing covariance functions and carrying out estimation and prediction.

## Appendix K. GTEx Data

The GTEx data can be downloaded from the GTEx portal: https://gtexportal.org/home/datasets. We use 52 tissue types, listed below, although some experiments use a subset of these tissue types. The sample size for each tissue type is shown in square brackets. Adipose Subcutaneous [644], Adipose Visceral (Omentum) [539], Adrenal Gland [254], Artery Aorta [424], Artery Coronary [238], Artery Tibial [657], Bladder [21], Brain Amygdala [147], Brain Anterior cingulate cortex (BA24) [172], Brain Caudate (basal ganglia) [230], Brain Cerebellar Hemisphere [208], Brain Cerebellum [241], Brain Cortex [255], Brain Frontal Cortex (BA9) [200], Brain Hippocampus [188], Brain Hypothalamus [193], Brain Nucleus accumbens (basal ganglia) [232], Brain Putamen (basal ganglia) [194], Brain Spinal cord (cervical c-1) [155], Brain Substantia nigra [133], Breast Mammary Tissue [456], Cells Cultured fibroblasts [444], Cells EBV-transformed lymphocytes [174], Cervix Ectocervix [9], Cervix Endocervix [10], Colon Sigmoid [373], Colon Transverse [406], Esophagus Gastroe-sophageal Junction [375], Esophagus Mucosa [551], Esophagus Muscularis [515], Fallopian Tube [9], Heart Atrial Appendage [422], Heart Left Ventricle [428], Kidney Cortex [85], Kidney Medulla [4], Liver [224], Lung [573], Minor Salivary Gland [162], Nerve Tibial [615], Ovary [180], Pancreas [37], Pituitary [283], Prostate [245], Skin Not Sun Exposed (Suprapubic) [595], Skin Sun Exposed (Lower leg) [665], Small Intestine Terminal Ileum [187], Spleen [233], Stomach [359], Testis [359], Uterus [142], Vagina [156], Whole Blood [139].
