# Supplementary material for: Bayesian Multi-Group Gaussian Process Models for Heterogeneous Group-Structured Data
Source: J Mach Learn Res. Author manuscript; Available in PMC 2025 Sep 26. (PMC12463451)
Supplement: 1 [file NIHMS2107171-supplement-1.pdf]

Appendix L. Supplementary figures

copy.png

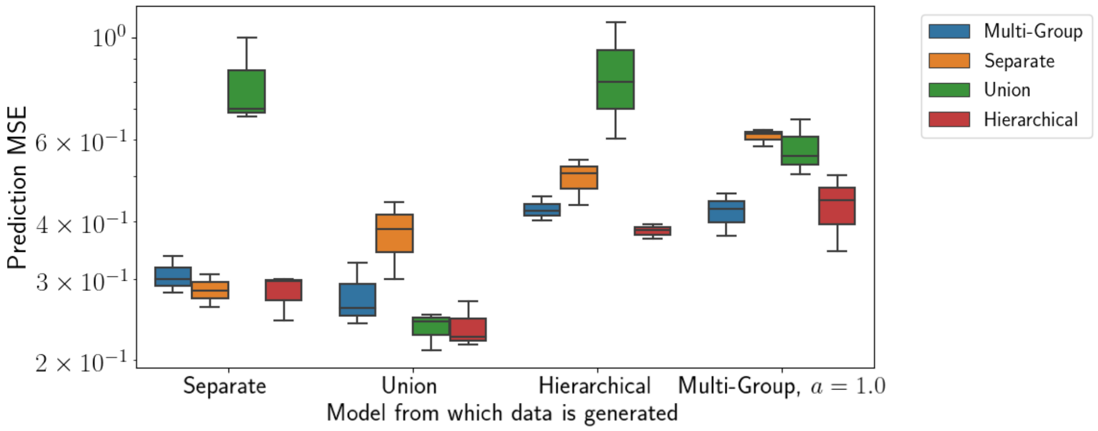

Figure 9: **Prediction experiment with synthetic data using a Matérn covariance function, as described in Section 4.2.** The covariance function parameters were set as $\sigma^2 = b = 1$, $\nu = 1/2$.

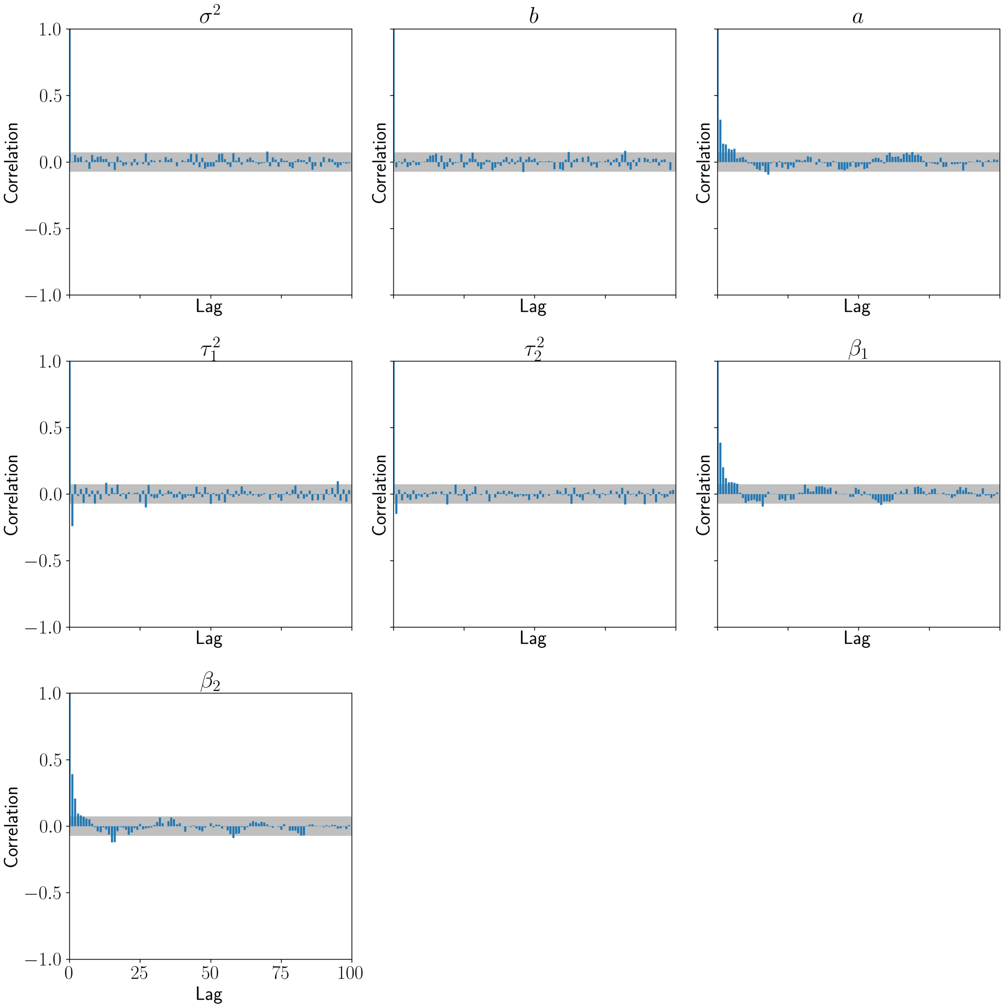

Figure 10: **Autocorrelation plots for MCMC samples from Multi-Group process model's posterior distribution, as described in Section 4.2. Gray bands cover** ± 0.05.

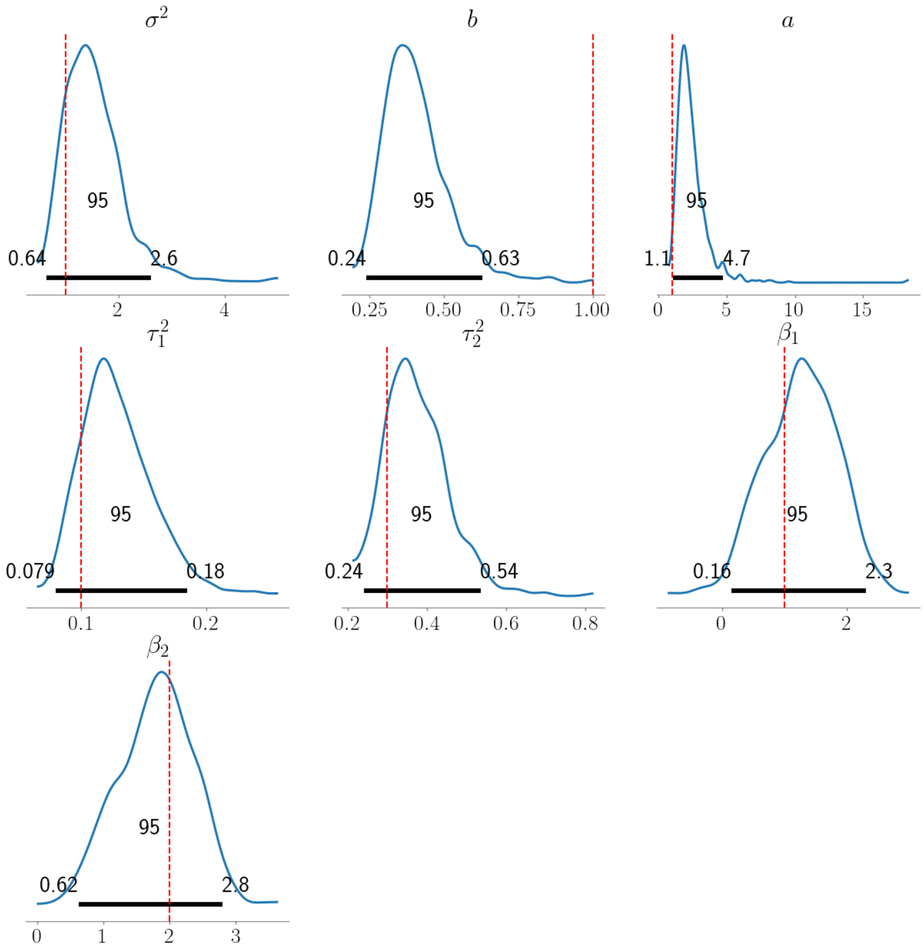

Figure 11: **Posterior samples of covariance function and model parameters from the Bayesian analysis described in Section 4.2**. Curves show the density of posterior samples for each parameter and black horizontal bars show the highest 95% density intervals for each set of samples. Red vertical lines indicate the parameter values used to generate the data.

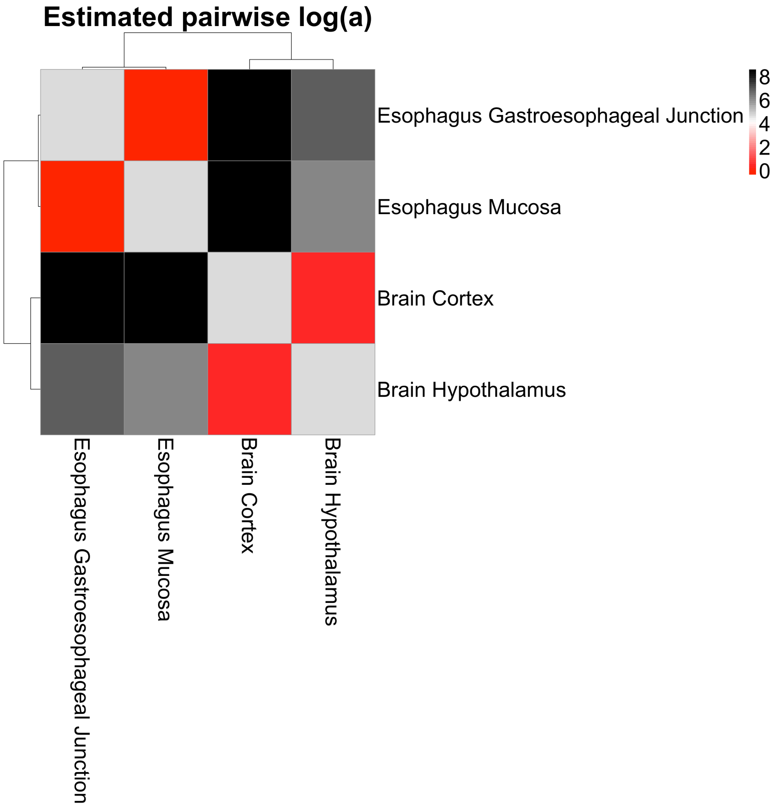

Figure 12: **Estimation of a for each pair of GTEx tissue types**. Cell ij in the heatmap represents $\log_{10}(a_{ij})$, where a_{ij} is the MLE of a when fitting the Multi-Group process using tissues i and j. Lower values of a (red) indicate higher similarity, while higher values of a (black) indicate lower similarity. Here, we allow each group its own noise variance τ_j^2.

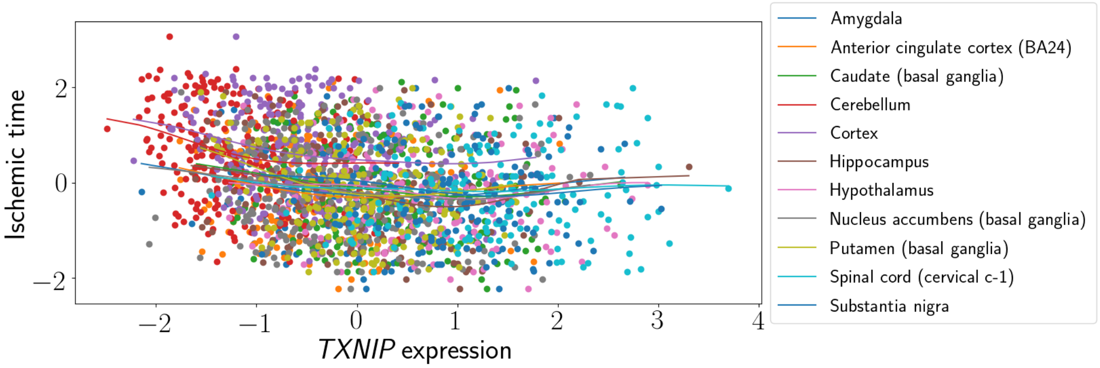

Figure 13: **Bayesian analysis of GTEx data across all brain tissues.** Points show the *TXNIP* expression and ischemic time for brain tissues. Each line shows the mean of the group-specific predictive process estimated using the MGGP.

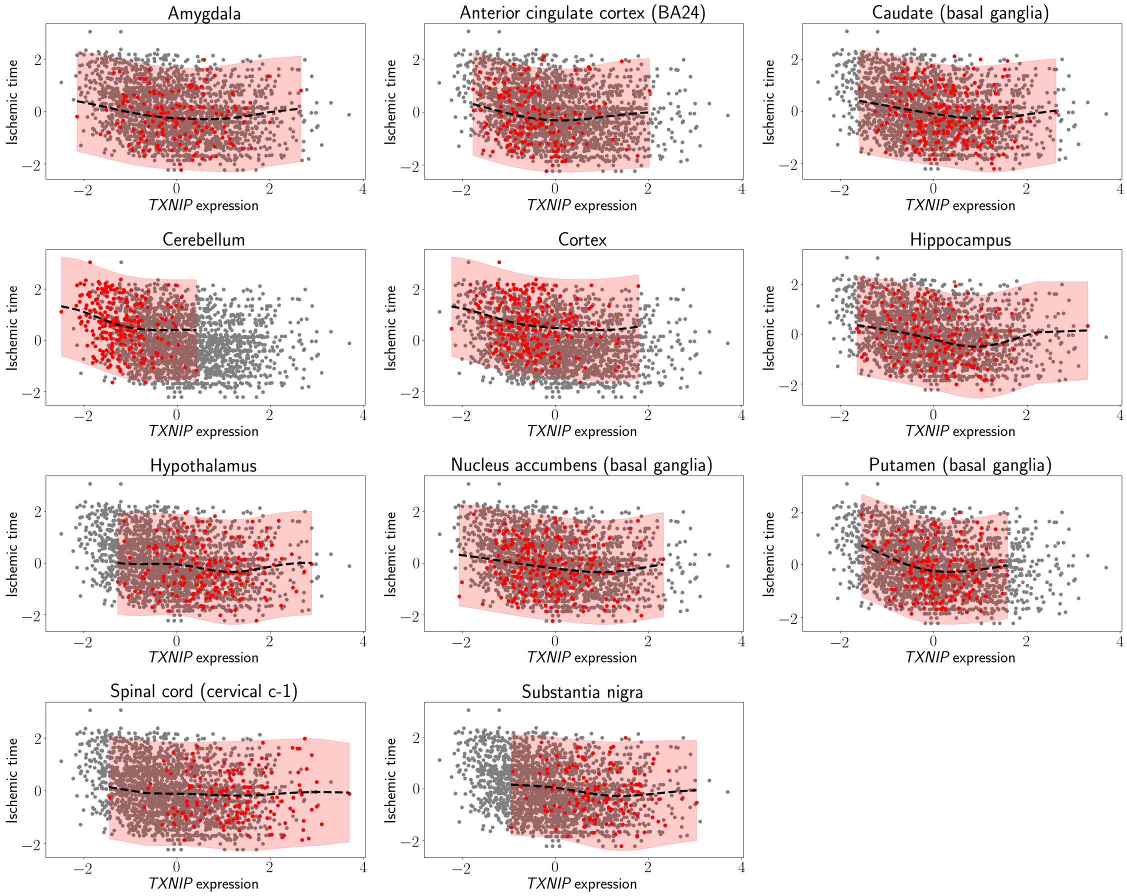

Figure 14: **Bayesian analysis of GTEx data for each brain tissue.** Points belonging to each group are colored in red. Points show the *TXNIP* expression and ischemic time for brain tissues. The dashed line in each panel shows the mean of the group-specific predictive process estimated using the MGGP, and the bands cover twice the standard deviation above and below the mean.
